# The cytosolic Fe-S cluster assembly component MET18 is required for the full enzymatic activity of ROS1 in active DNA demethylation

**DOI:** 10.1038/srep26443

**Published:** 2016-05-19

**Authors:** Xiaokang Wang, Qi Li, Wei Yuan, Zhendong Cao, Bei Qi, Suresh Kumar, Yan Li, Weiqiang Qian

**Affiliations:** 1 State Key Laboratory of Protein and Plant Gene Research, The Peking-Tsinghua Center for Life Sciences, School of Advanced Agricultural Sciences and School of Life Sciences, Peking University, Beijing 100871, China; 2 Division of Biochemistry, Indian Agricultural Research Institute, New Delhi 110012, India

## Abstract

DNA methylation patterns in plants are dynamically regulated by DNA methylation and active DNA demethylation in response to both environmental changes and development of plant. Beginning with the removal of methylated cytosine by ROS1/DME family of 5-methylcytosine DNA glycosylases, active DNA demethylation in plants occurs through base excision repair. So far, many components involved in active DNA demethylation remain undiscovered. Through a forward genetic screening of *Arabidopsis* mutants showing DNA hypermethylation at the *EPF2* promoter region, we identified the conserved iron-sulfur cluster assembly protein MET18. MET18 dysfunction caused DNA hypermethylation at more than 1000 loci as well as the silencing of reporter genes and some endogenous genes. MET18 can directly interact with ROS1 *in vitro* and *in vivo*. ROS1 activity was reduced in the *met18* mutant plants and point mutation in the conserved Fe-S cluster binding motif of ROS1 disrupted its biological function. Interestingly, a large number of DNA hypomethylated loci, especially in the CHH context, were identified from the *met18* mutants and most of the hypo-DMRs were from TE regions. Our results suggest that MET18 can regulate both active DNA demethylation and DNA methylation pathways in *Arabidopsis*.

Epigenetic modifications are important for the silencing of transposable element (TE), chromosome stability, gene expression and many other vital cellular processes^1^,^2^,^3^. DNA methylation is an important epigenetic modification conserved in plants and other higher eukaryotes. Unlike the situation in mammals, wherein DNA methylation predominantly occurs in the CG context^4^, it occurs in all three cytosine contexts: CG, CHG, and CHH (H= A, T, or C) in plants. Whole-genome bisulfite sequencing in *Arabidopsis* has revealed that gene bodies are mainly associated with CG methylation. While TE- and repeats-enriched heterochromatin regions are the major targets of CHG and CHH methylation, they are also densely CG methylated^5^. In *Arabidopsis*, once the DNA methylation pattern is established, symmetric CG and CHG methylation can be maintained by DNA METHYLTRANSFERASE 1 (MET1) and CHROMOMETHYLASE 3 (CMT3) during DNA replication, while asymmetric CHH methylation must be *de novo* established by DOMAINS REARRANGED METHYLASE 2 (DRM2) through an RNA-directed DNA methylation (RdDM) pathway^1^,^6^. In RdDM, two plant-specific RNA polymerases Pol IV and Pol V produce 24-nt siRNAs and long noncoding scaffold transcripts respectively to assist in the recruitment of DRM2 to specific loci^1^,^6^. In addition to DRM2, CHROMOMETHYLASE 2 (CMT2) was recently shown to be a methyltransferase that catalyzes CHH methylation^7^.

DNA demethylation may take place as a passive or an active process. In contrast to the passive demethylation during which DNA methylation is lost due to the inactivation or down-regulation of maintenance DNA methyltransferases (such as DNMT1 and MET1 in mammals and plants, respectively), active DNA demethylation refers to the enzymatic removal of the methylated cytosine in a replication-independent manner^8^. In plants, the active DNA demethylation pathway is initiated by a subfamily of atypical HhH-GPD enzymes including REPRESSOR OF SILENCING1 (ROS1)^9^, DEMETER (DME), DEMETER-LIKE2 (DML2) and DEMERTER-LIKE3 (DML3)^10^,^11^. All of these enzymes are bifunctional 5-methylcytosine-DNA glycosylases/lyases that not only remove the 5-methylated cytosine, but also cleave phosphodiester backbone at the abasic site^10^,^12^,^13^,^14^ resulting in a gap with 3′ phosphate or 3′ dRP (3′ α, β-unsaturated aldehyde) termini. A DNA phosphatase (ZDP) and an apurinic/apyrimidinic endonuclease (APE1L) process the 3′ phosphate and 3′ dRP termini, respectively, to generate a 3′ OH group so that downstream polymerases and ligases can fill in the gap with an unmethylated cytosine^15^,^16^. Interestingly, DNA demethylation in mammals also utilizes the BER pathway but with a different strategy at the beginning. Instead of being directly removed, in mammals the 5mC is converted into other bases such as 5-hydroxymethylcytosine (5hmC) by the TET family enzymes, which can be further oxidized to produce 5fC and 5caC. The 5fC or 5caC can then be removed by thymine DNA glycosylase (TDG)^8^,^17^.

Compared to the well-established RdDM pathway, little is currently known about the locus-specific guidance of active DNA demethylation machinery in plants. Previous studies have shown that the RNA-binding protein ROS3 is required for the recruitment of ROS1 to a subset of its target loci^18^,^19^. Recently, an IDM1-IDM2-IDM3-MBD7 complex was identified as another important regulator of DNA demethylation that functions upstream of ROS1^20^,^21^,^22^,^23^,^24^,^25^. The IDM1 complex recognizes methylated DNA loci with low H3K4 and H3R2 methylation levels and catalyzes H3K18 and H3K23 acetylation, which facilitates ROS1-mediated DNA demethylation^20^,^21^,^24^,^26^. Unlike ROS1, which functions in almost all plant tissues, DME is preferentially expressed in the central cell of the female gametophyte. Active DNA demethylation induced by DME in the central cell is essential for the expression of imprinting genes, including *FWA* and *MEA*^11^. In addition to DME, mutations in two cytosolic iron-sulfur cluster assembly (CIA) components NAR1 and DRE2 also influence the expression of *FWA*^27^,^28^.

Many proteins that function in vital processes such as respiration, photosynthesis, sulfur and nitrogen assimilation, amino acid and purine metabolism, plant hormone and coenzyme synthesis, DNA repair and translation have Fe-S clusters as their cofactors^29^,^30^. In plants, there are three Fe-S cluster assembly pathways: the SUF (sulfur mobilization) pathway in plastids, the ISC (iron-sulfur cluster) assembly pathway in mitochondria and an emerging CIA pathway in the cytoplasm that is partially dependent on the ISC pathway^29^,^30^,^31^. The CIA pathway, mainly found in eukaryotes, is essential for the maturation of both cytoplasmic and nuclear Fe-S proteins^32^. In yeast, a mitochondrial cysteine desulfurase complex Nfs1-Isd11 and the mitochondrial ISC machinery initially synthesize a sulfur-containing component, which is then exported to the cytoplasm by the mitochondrial inner membrane ATP-binding cassette transporter Atm1^30^,^33^,^34^. In the cytoplasm of yeast and human cells, Fe-S clusters are assembled on the CFD1-NBP35 heterodimer whereas in plants Fe-S clusters are assembled on the NBP35 homodimer^35^. Fe-S clusters are then transferred to the apoproteins, with the help of the NAR1 and CIA targeting complex comprised of a WD40-repeat protein CIA1, a small acidic protein CIA2 and a HEAT-repeat containing protein MET18/MMS19^30^,^36^,^37^,^38^. Recent findings suggest that MET18/MMS19 is a late-acting CIA component^30^,^38^,^39^. In yeast, MET18/MMS19 also forms a complex with Dos2, Rik1 and Cdc20, and participates in DNA repair, RNA polymerase II transcription, chromosome segregation and telomere length maintenance^40^,^41^,^42^,^43^,^44^. Since the ROS1/DME family members are Fe-S proteins^29^,^45^,^46^, a deficiency in the CIA pathway may have impact on the activities of ROS1/DME family members.

Here we report MET18 as a component of the active DNA demethylation pathway in plants. Identified through a forward genetic screening, the *met18* mutant plants exhibited DNA hypermethylation at hundreds of genetic loci and conferred transcriptional silencing of reporter genes and some endogenous targets. Most of the hyper-DMRs (differentially methylated regions) identified from the *met18* mutants were also hypermethylated in the *ros1-4* or *rdd* (*ros1dml1dml2* triple) mutant plants. Interaction between MET18 and ROS1 was observed *in vitro* as well as *in vivo*. ROS1 activity was reduced in the *met18* mutants. Interestingly, we found that dysfunction of MET18 caused DNA hypomethylation and the activation of some of the TEs. Our results revealed an epigenetic role of MET18 in the regulation of gene expression in *Arabidopsis*.

## Results

### Isolation of *met18* mutants

**Figure 1:**
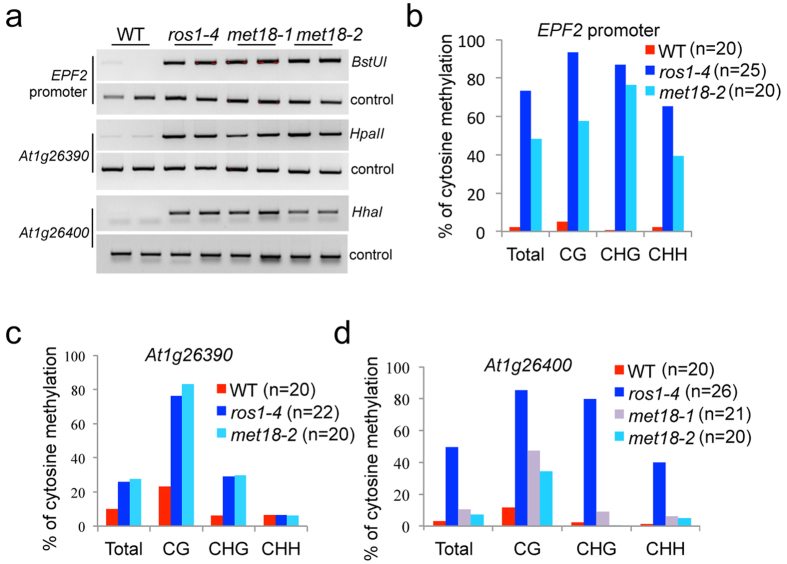
Dysfunction of *MET18* causes DNA hypermethylation at the *EPF2* promoter, *At1g26390* and *At1g26400* loci. (**a**) Analysis of DNA methylation levels at the *EPF2* promoter, *At1g26390* and *At1g26400* loci using chop-PCR. The methylation-sensitive restriction enzymes used were *Bst*UI, *Hpa*II and *Hha*I. DNA hypermethylation results in no cleavage by the enzymes and increased levels of the PCR product. Undigested controls are shown in the lower panel. (**b**–**d**) Bisulfite sequencing data showing the effects of *MET18* mutations on DNA methylation in different sequence contexts in the *EPF2* promoter (**b**), *At1g26390* (**c**) and *At1g26400* (**d**). For each sample, at least 20 clones were analyzed and the percentage of DNA methylation was calculated from the indicated number of clones.

Our previous studies have shown that IDM1 is crucial for the recruitment of ROS1 to only a subset of its targeted loci^20^, suggesting that there exists an IDM1-independent pathway to regulate ROS1-mediated active DNA demethylation in *Arabidopsis*. To identify new components in the active DNA demethylation pathway initiated by ROS1, a new chop-PCR marker was designed based on the DNA methylation analysis at the *EPF2* promoter (Supplementary Fig. 1a). The chop-PCR analysis of the *EPF2* promoter revealed that the DNA methylation level in this region was significantly higher in the *ros1-4* and *rdd* mutants but not in the *idm1* and *idm2* mutants, which was consistent with the whole-genome bisulfite sequencing results (Supplementary Fig. 1b). The expression level of *EPF2* was reduced in *ros1-4* but not in *idm1-1* (Supplementary Fig. 1c). Screening of the *Arabidopsis* T-DNA insertion mutants library, which includes mutants of chromatin-related genes, DNA or RNA binding protein-encoding genes and genes co-expressed with *ROS1* or *ROS3 et al.* (Supplementary Table 1), using this chop-PCR marker resulted in the identification of two mutants showing DNA hypermethylation at the *EPF2* promoter region. One of them harbors a T-DNA insertion in the *At5g48120* gene and is referred to here as *met18-1* (Fig. 1a). To confirm that the DNA hypermethylation was due to the T-DNA insertion mutation, we tested another mutant allele (i.e. *met18-2*). As expected, *met18-2* showed a similar DNA hypermethylation phenotype in the *EPF2* promoter region (Fig. 1a). Locus-specific bisulfite sequencing of the *EPF2* promoter showed that the DNA methylation levels were increased in *met18-2* and *ros1-4* in all sequence contexts when compared to the levels in the wild-type (WT) control (Fig. 1b). Examination of the methylation status at previously identified marker genes *At1g26390* and *At1g26400* revealed that both *met18* mutants were hypermethylated at these loci in all sequence contexts (Fig. 1a,c,d)^20^. To further confirm that *MET18* is the gene responsible for the DNA hypermethylation phenotype, the *met18-1* mutant plants were transformed with an 8.5 kb WT genomic fragment containing the promoter and coding sequence of the *At5g48120* gene. The transgene complemented the DNA hypermethylation phenotype of *met18-1* (Supplementary Fig. 1d,e). All of these findings suggest that MET18 is a new component of the active DNA demethylation pathway.

### MET18 prevents DNA hypermethylation at hundreds of loci

**Figure 2:**
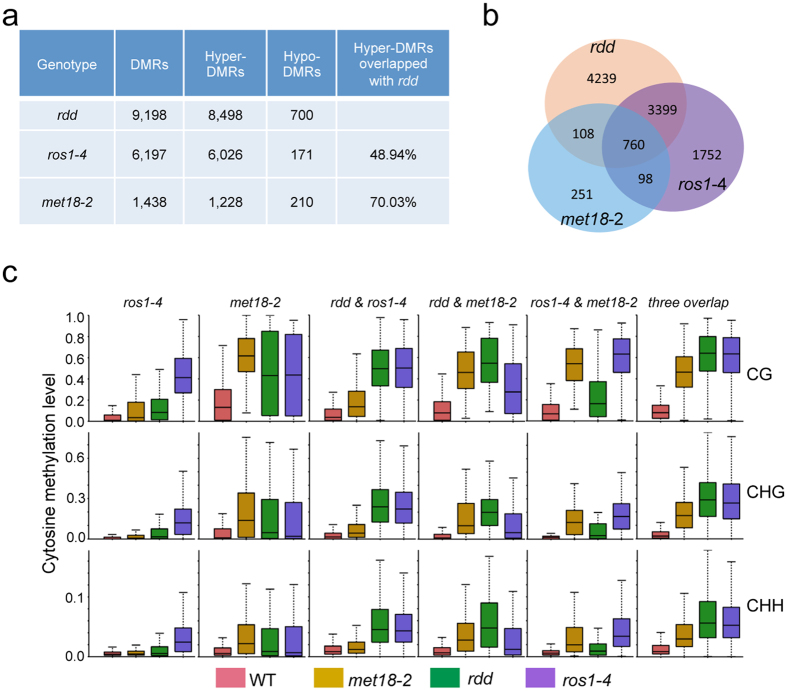
Dysfunction of *MET18* causes genome-wide DNA hypermethylation. (**a**) The number of differentially methylated regions (DMRs) identified in *met18-2* and the overlapping DMRs between different mutant plants. (**b**) Venn diagram showing the numbers of hyper-DMRs that either overlap between or are unique in *met18-2*, *ros1-4*, and *rdd.* (**c**) Boxplots showing the distribution and the average CG, CHG, or CHH methylation levels calculated from DMRs in the respective subgroups. Dark horizontal line, median; edges of boxes, 25th (bottom) and 75th (top) percentiles; whiskers, minimum and maximum percentage of DNA methylation.

In order to examine the role of MET18 in active DNA demethylation, we compared the genome-wide DNA methylation profiles of *met18-2* and WT Col-0 plants by next-generation sequencing after bisulfite conversion^4^,^20^. There was no significant difference in the overall genome methylation patterns between *met18-2* and WT plants. However, mutation in *MET18* caused DNA hypermethylation at 1,228 loci and hypomethylation at 210 loci. The differences between *met18-2* and WT plants in DNA methylation at these loci are statistically significant with the Benjamini–Hochberg adjusted *p* value less than 0.01 (Fig. 2a and Supplementary Table 2). Consistent with the locus-specific bisulfite sequencing results, methylome analysis also showed hypermethylation of *At1g26390* and *At1g26400* in *ros1-4* and *met18-2* (Supplementary Fig. 2a,b)^20^. Analysis of the distribution of the hypermethylated loci on all of the five chromosomes in *met18-2*, *ros1-4* and *rdd* indicated that the hypermethylated loci in *met18-2* were spread evenly over the five chromosomes and that they largely overlapped with the hypermethylated loci in *ros1-4* and *rdd* (Supplementary Fig. 2c). Analysis of the hyper-DMRs in different DNA regions (gene body, intergenic region, TEs out of gene region and TEs overlapping with gene region) showed a similar pattern between *met18*-2 and *ros1-4*, with less than 20% of the hyper-DMRs distributed in the gene body (Supplementary Fig. 2d).

About 70% of the 1,228 hyper-DMRs in *met18-2* were also hypermethylated in *ros1-4* and *rdd* (Fig. 2a,b). The overlapping hypermethylated loci in these mutants were methylated in all sequence contexts (Fig. 2c and Supplementary Fig. 2e). Notably, the DNA methylation level at the *met18-2*-specific loci was actually also higher in *ros1-4* and *rdd* (Fig. 2c and Supplementary Fig. 2e), although the increases were not significant enough to be counted as hyper-DMRs as per the parameters defined in this study. These results indicated that MET18 and ROS1 might be functional in the same genetic pathway of active DNA demethylation. However, no increase in the DNA methylation level at the *ros1-4-* or *rdd*-specific loci was observed in *met18-2* (Fig. 2c), suggesting that MET18 may only control DNA demethylation at a subset of ROS1 targeted loci. To further determine whether MET18 regulates DNA demethylation in an IDM1-independent manner, we compared the hyper-DMRs identified from *met18-2* and *idm1-1*. Our results showed that 181 hyper-DMRs overlapped between them, accounting for only 14.7% of the hyper-DMRs in *met18-2* and 16.4% of the hyper-DMRs in *idm1-1* (Supplementary Fig. 2f). The low level of hyper-DMR overlap between these two mutants suggests that they may act through different pathways.

### MET18 prevents the silencing of reporter genes and some endogenous genes

**Figure 3:**
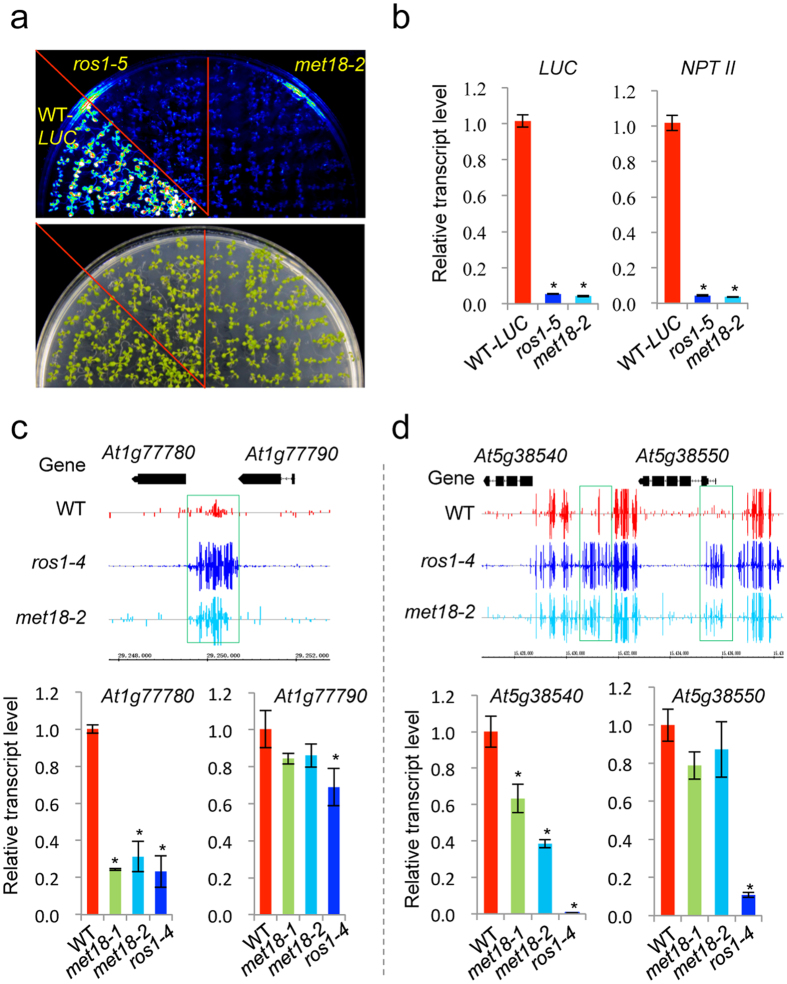
MET18 prevents the silencing of reporter genes and endogenous genes. (**a**) The *MET18* mutation causes the silencing of *35S-LUC* and *35S-NPTII* reporter genes. The reporter genes were introduced into *met18-2* by crossing. Seedlings grown in MS plates were imaged after being sprayed with luciferase substrate. (**b**) Real-time PCR analysis of the expression levels of the *LUC* and *NPTII* reporter genes in the different genotypes. (**c**) Effect of *MET18* mutation on the DNA methylation levels in WT control, *ros1-4* and *met18-2*, and the expression levels of endogenous genes *At1g77780* and *At1g77790.* (**d**) Effect of *MET18* mutation on the DNA methylation levels in WT control, *ros1-4* and *met18-2*, and the expression levels of endogenous genes *At5g38540* and *At5g38550* in the mutants. Snapshots in the Integrated Genome Browser show the DNA methylation levels, while the bar diagrams show the real-time PCR analysis of the expression levels of the hypermethylated genes or nearby genes in different genotypes. *TUB8* was used as an internal control. Standard errors were calculated from three biological replications, *P < 0.05.

Previous studies have reported that ROS1 was required for the anti-silencing of reporter genes and endogenous genes^9^. We introduced *35S::LUC* and *35S::NPT II* transgenes into the *met18-2* background via crossing, and we found that both *LUC* and *NPT II* were silenced in *met18-2* (Fig. 3a,b). We also examined the expression levels of twelve endogenous genes by using real-time PCR to determine whether DNA hypermethylation affects their expression in the *met18* mutants. Nine of the tested genes showed a significant reduction in their transcript levels in the *met18* mutants (Fig. 3c and Supplementary Fig. 3). Unexpectedly, the expression level of *EPF2* was not reduced in the *met18* mutants in spite of the fact that we did identify the *met18* mutants via chop-PCR using the *EPF2* marker (Supplementary Fig. 3a). Bisulfite sequencing results indicated that the increase in DNA methylation in the *EPF2* promoter in *met18-2* was not as high as that observed in *ros1-4*, suggesting that the increase in DNA methylation in the *met18* mutants may not be sufficient to silence the *EPF2* gene (Supplementary Fig. 3a). To further explore the anti-silencing role of MET18 genome-wide, we analyzed previously published RNA-seq data generated using *met18-2*^47^. However, we only identified 4 genes (*AT1G48660, AT1G77960, AT3G26742* and *AT3G27940*), out of 132 down-regulated genes in *met18-2*, that are hypermethylated (Supplementary Table 2). Our analysis suggests that most of the down-regulated genes are not controlled by MET18 directly through DNA methylation.

### MET18 is a component of the CIA complex

To investigate the role of MET18 in the regulation of active DNA demethylation, the protein complex associated with MET18 was purified from floral tissues of the *met18-1* plants harboring *MET18-3Flag* transgene driven by native *MET18* promoter, which could complement the DNA methylation phenotype (Supplementary Fig. 1d,e). As expected, the most abundant peptides identified by the mass spectrometric analysis corresponded to MET18. Meanwhile, several other components of the CIA complex, including CIA1, CIA2/AE7, NAR1 and AtDRE2 were also identified (Supplementary Tables 4 and 5). The peptides from CIA1 and CIA2/AE7 were much more abundant than those from NAR1 and AtDRE2, suggesting that MET18, CIA2/AE7 and CIA1 are major components of the CIA targeting complex and that the ratio for these three proteins should be 1:1:1 in the complex (Supplementary Table 5). Moreover, many Fe-S cluster proteins including ROS1, DME, and ACO1 were co-purified with MET18, suggesting the interaction between MET18 and these proteins (Supplementary Table 5). Taken together, our results suggest that as in mammals and yeast^38^,^39^, MET18 is also a component of the CIA complex in plants. Our results are consistent with the findings of two previous studies done by Luo *et al.*^45^ and Han *et al.*^47^.

### MET18 interacts with ROS1 *in vitro* and *in vivo*

**Figure 4:**
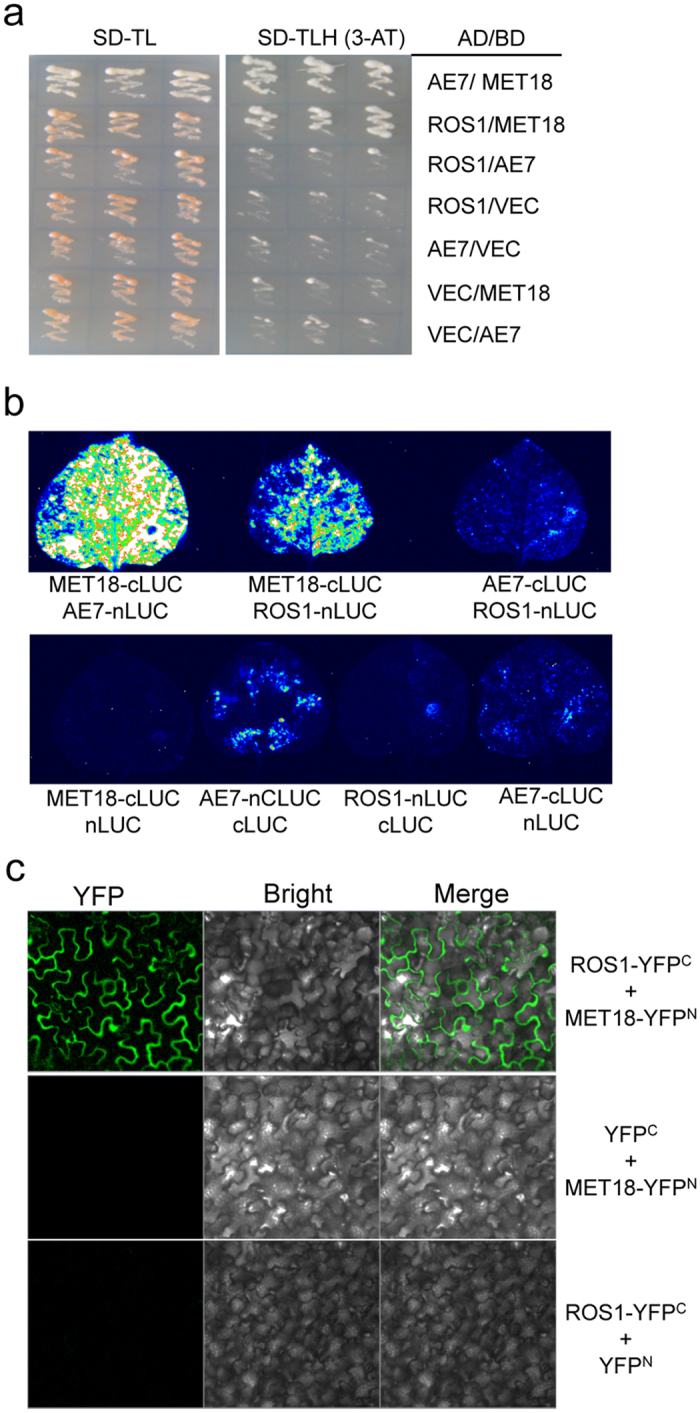
MET18 can interact with ROS1 *in vivo*. (**a**) Interaction of MET18 with ROS1 as determined by Y-2-H assay. Yeast cells harboring different fusion protein combinations (listed on the right) in pGBK-T7 (BD) and pGAD-T7 (AD) vectors were plated on the medium lacking Leu, Trp (SD-TL) or the medium lacking Leu, Trp and His (SD-TLH). Interaction between BD-MET18 and AD-AE7 serves as a positive control. (**b**) Interaction of MET18 with ROS1 as demonstrated by firefly luciferase complementation imaging assay in *Nicotiana benthamiana* leaves. Interaction between MET18-cLUC and AE7-nLUC serves as a positive control. nLUC, C-terminal region of firefly luciferase; cLUC, N-terminal region of firefly luciferase. (**c**) BiFC assay showing ROS1 and MET18 interaction in the cytoplasm. YFP^C^, C-terminal region of YFP; YFP^N^, N-terminal region of YFP. The experiments were repeated three times.

ROS1 and DME were co-purified with MET18, suggesting that MET18 interacts with these proteins (Supplementary Table 5). Since MET18 functions as a platform to facilitate Fe-S cluster transfer to targeted proteins in the CIA pathway in Arabidopsis^45^, and as ROS1 contains a Fe-S cluster binding motif (Supplementary Fig. 4), we speculated that MET18 physically interacts with ROS1. To detect such a physical interaction, we performed a yeast two-hybrid (Y-2-H) assay. The results showed that ROS1 can directly interact with MET18 but not with AE7, another component of the CIA complex (Fig. 4a). The *in vivo* interaction of MET18 and ROS1 was validated by a luciferase complementation imaging assay wherein the co-infiltration of tobacco leaves with MET18-cLUC and AE7-nLUC or MET18-cLUC and ROS1-nLUC activated the expression of the luciferase reporter gene and transmitted fluorescence (Fig. 4b).

ROS1 has been reported to be localized in the nucleus^9^, while MET18 was proposed to be a component of the cytosolic CIA complex in eukaryotes^38^,^39^,^45^, although it was also detected in the nucleus^40^,^45^. To examine where ROS1 and MET18 interact *in vivo*, we first examined the subcellular localization of MET18 using stably expressing *MET18-3Flag* transgenic plants. Nuclear and cytoplasmic proteins extracted from the transgenic plants were subjected to Western blot analysis using HSP90 and histone H3 as markers for the cytoplasmic proteins and nuclear proteins, respectively. The results indicated that MET18 was specifically localized in the cytoplasm but not in the nucleus (Supplementary Fig. 5). Secondly, to check for interaction between ROS1 and MET18, we performed a bimolecular fluorescence complementation (BiFC) assay. It primarily detected a YFP signal in the cytoplasm (Fig. 4c), suggesting that ROS1 and MET18 interact mainly in the cytoplasm.

### MET18 is required for ROS1 activity

**Figure 5:**
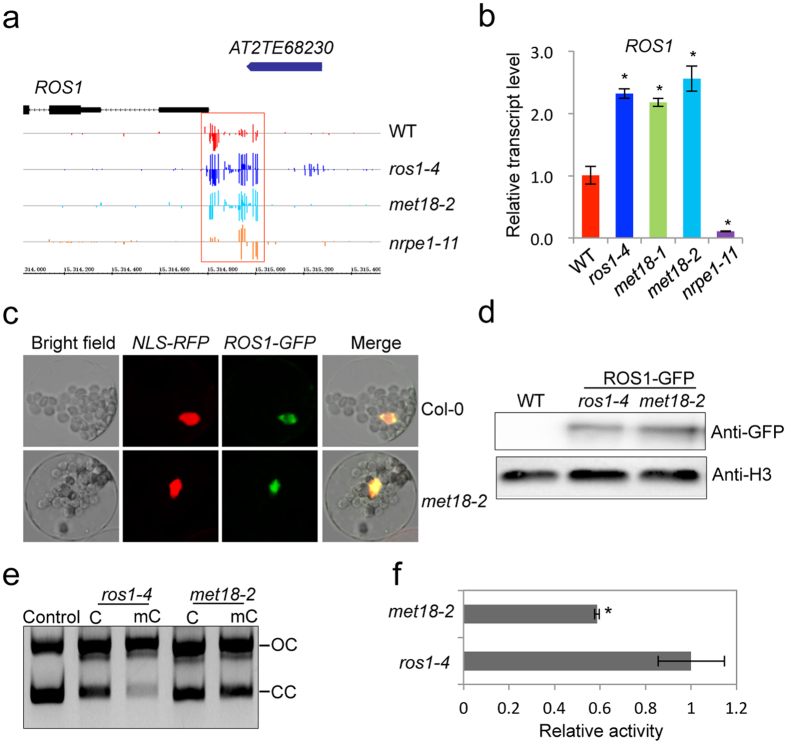
Dysfunction of MET18 affects ROS1 activity. (**a**) Snapshots in the Integrated Genome Browser showing the DNA methylation levels at the *ROS1* promoter. The specific region important for *ROS1* regulation is highlighted with red box. (**b**) Real-time PCR analysis of *ROS1* transcripts in different mutants. Standard errors were calculated from three biological repeats, *P < 0.05. (**c**) Localization of ROS1-GFP in the protoplasts of *met18-2*. NLS-RFP was used as a control for nuclear localized protein. ROS1-GFP and NLS-RFP constructs were co-introduced into the Col-0 or *met18-2* mutant protoplasts. GFP signal (green) and overlay of the signals from GFP and RFP (red) are shown. (**d**) Detection of ROS1-GFP protein by GFP antibody. Anti-H3 serves as a loading control. (**e,f**) Nicking activity of ROS1-GFP fusion protein from *met18-2* and *ros1-4* transgenic plants. OC, open circular form plasmid DNA; CC, closed circular plasmid DNA. Standard errors were calculated from three biological replications, *P < 0.05.

ROS1 function and RdDM pathway have recently been found to be tightly interconnected^48^,^49^. In the RdDM pathway mutants, the *ROS1* promoter is hypomethylated and the transcript level of *ROS1* is dramatically reduced^48^,^49^. By contrast, in the *ros1* and *rdd* mutants, the *ROS1* promoter is hypermethylated and the transcript level of *ROS1* is increased^48^,^49^. Our whole-genome bisulfite sequencing results and real-time PCR analysis showed that *met18-2* was similar to *ros1-4* in their methylation levels at *ROS1* promoter and their *ROS1* transcript levels (Fig. 5a,b), suggesting that the DNA hypermethylation in *met18* was not due to decreased ROS1 transcription. As a key component of the CIA pathway, MET18 is essential for the stability of the targeted proteins^38^. Hence, we investigated whether the loss of MET18 led to DNA hypermethylation through affecting the localization or stability of ROS1 in the nucleus. To this end, we transiently expressed ROS1-GFP fusion protein in *ros1-4* or *met18-2* protoplasts. We observed that ROS1 was localized exclusively in the nucleus both in *met18-2* and *ros1-4* (Fig. 5c). Meanwhile, we conducted Western blot using the GFP antibody and our results confirmed that *MET18* mutation did not affect the stability of ROS1 protein (Fig. 5d). These results suggest that *MET18* mutation did not affect the localization or the stability of ROS1 protein. We then compared the nicking activity of ROS1 protein on methylated DNA using immunoprecipitated ROS1-GFP fusion protein from *met18-2* and *ros1-4*. Our results showed that the nicking activity of ROS1-GFP protein purified from *met18-2* was reduced compared with that from *ros1-4* (Fig. 5e,f), suggesting that *MET18* mutation mainly affected active DNA demethylation by compromising the enzymatic activity of ROS1 and ROS1 family proteins.

### The iron-sulfur cluster binding motif is essential for ROS1 function

**Figure 6:**
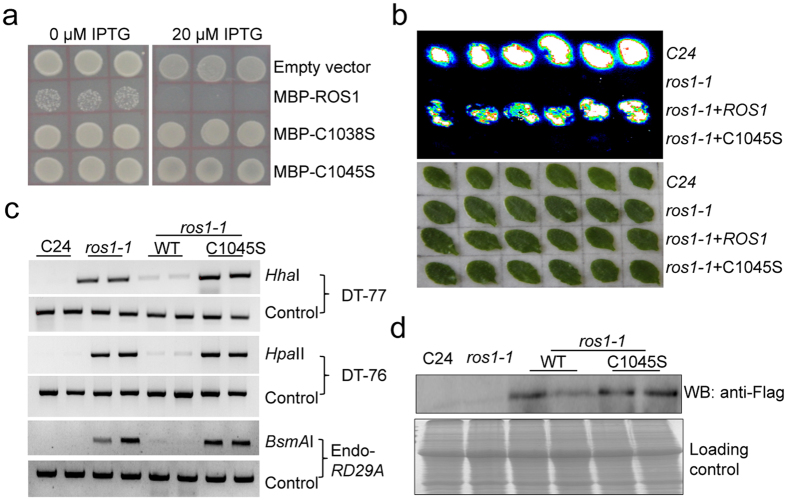
Iron-Sulfur cluster binding site is essential for ROS1 function. (**a**) Enzymatic activity of WT and mutated ROS1 in *E. coli* indicated by colony formation of WT and mutated (C1038S and C1045S) ROS1 transformants at 0 and 20 μM IPTG. (**b**) Complementation assay showing that WT, but not the mutant form of ROS1-3Flag, can restore *LUC* expression in *ros1-1*. Leaves were collected for luminescence imaging after the treatment with 3% NaCl for 6 h. (**c**) DNA hypermethylation phenotype of *ros1-1* plant transformed with the WT or mutant form (C1045S) of *ROS1*. Methylation-sensitive restriction enzymes (*Hha*I, *Hpa*II and *BsmA*I) were used for the chop-PCR analysis and undigested DNA was amplified as a control. (**d**) Detection of WT ROS1 protein and site-directed mutated (C1045S) ROS1 protein in transgenic *Arabidopsis* plants by Flag antibody. About 10 μg protein extracts were loaded in each well. The gel for loading control was stained with Coomassie blue.

ROS1/DME family members possess four conserved cysteine residues adjacent to the DNA glycosylase domain that may function to bind the [4Fe-4S] cluster (Supplementary Fig. 4). Any cysteine mutation in DME protein has been reported to cause the loss of 5mC excision activity *in vitro*^46^, suggesting that the iron-sulfur binding motifs are essential for the enzymatic activity of the ROS1/DME family proteins. To investigate whether the binding of the Fe-S cluster has any impact on the activity of ROS1, we introduced C1038S and C1045S mutations in the conserved iron-sulfur cluster binding motif of ROS1 via site-directed mutagenesis (Supplementary Fig. 4). Our Split-LUC assay showed that the mutations didn’t affect the interaction between ROS1 and MET18 (Supplementary Fig. 6). We further tested whether these mutations affect ROS1 activity by measuring the viability of *E.coli* cells expressing WT or mutated ROS1. Since a previous study demonstrated that the expression of active DME induces lethality in methylation-proficient *E.coli*^46^, we speculated that the expression of other members of the demethylating DNA glycosylase family, including ROS1, may also cause lethality in *E.coli* and measuring *E.coli* viability could be a way to determine whether ROS1 is active. Indeed we found that the expression of WT ROS1 caused lethality in *E.coli*, but the expression of C1038S or C1045S mutant form of ROS1 had no effect on the viability of *E.coli* (Fig. 6a), suggesting that C1038S or C1045S mutation abolished the enzymatic activity of ROS1 protein. Moreover, we found that the expression of WT, but not the C1045S mutant version of ROS1, under a constitutive promoter could complement the luciferase and DNA hypermethylation phenotypes of the *ros1-1* mutant (Fig. 6b,c), despite equal amounts of WT and mutated ROS1 being expressed (Fig. 6d). These results suggest that the Fe-S cluster binding motif is crucial for ROS1 function *in vivo*.

### Mutation in MET18 causes DNA hypomethylation in the CHH context at TE regions

**Figure 7:**
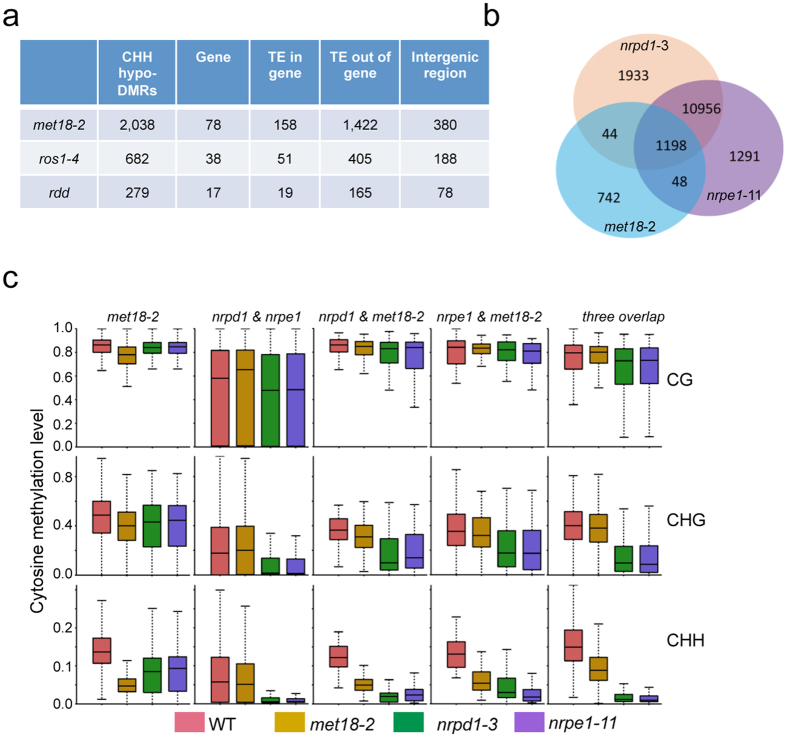
Comparison of CHH hypo-DMRs among *met18-2*, *nrpd1-3*, and *nrpe1-11* mutants. (**a**) Number and location of CHH hypo-DMRs identified in *met18-2* and other mutant plants. (**b**) Venn diagram showing the numbers of CHH hypo-DMRs that either overlap between or are unique in *met18-2*, *nrpd1-3*, and *nrpe1-11*. (**c**) Boxplots showing the distribution and the average CG, CHG and CHH methylation levels calculated from hypo-DMRs in the respective subgroups. Dark horizontal line, median; edges of boxes, 25th (bottom) and 75th (top) percentiles; whiskers, minimum and maximum percentage of DNA methylation.

Although the overall DNA methylation level in the genic regions of *met18-2* and WT control was similar, methylation in the CHH context in TEs was slightly reduced in *met18-2* while it was slightly increased in *ros1-4* or *rdd* (Supplementary Fig. 7a–c). CHH hypomethylation of 2038 loci was observed in *met18-2* compared to a significantly lower number of hypomethylated loci in *ros1-4* and *rdd* (Fig. 7a and Supplementary Table 3). Examination of the effect of *MET18* mutation on DNA methylation in the CHH context in TEs of different lengths revealed that *MET18* mutation had a larger impact on shorter TEs (Supplementary Fig. 7d). Distribution analysis of the CHH hypo-DMRs on all of the five chromosomes in *met18-2* indicated that the CHH hypo-DMRs were enriched at the pericentromeric regions (Supplementary Fig. 8a). Most of the CHH hypo-DMRs were from TE regions and more than half of them were overlapped with CHH hypo-DMRs in the Pol IV mutant (*nrpd1-3*) and Pol V mutant (*nrpe1-11*) (Fig. 7a,b). We also examined the methylation level in *nrpd1-3* and *nrpe1-11* at those regions that were hypomethylated in *met18-2* in the CHH context and found that most of them were hypomethylated, albeit to different degrees (Supplementary Fig. 8b). The overlapping loci in these mutants were hypomethylated in the CHH context (Fig. 7c). Methylation in the CHG context was also reduced in *nrpd1-3* and *nrpe1-11*, but not in *met18-2* for the three overlapped loci (Fig. 7c). DNA methylation at the *met18-2*-specific loci was also decreased in *nrpd1-3* and *nrpe1-11* (Fig. 7c) although the DNA methylation levels at the *nrpd1-* and *nrpe1*-specific loci were not decreased in *met18-2*. Taken together, our results indicated that MET18 may also regulate DNA methylation pathway.

### Dysfunction of MET18 causes impaired production of 24-nt siRNAs and long noncoding scaffold RNAs as well as abolished transcriptional silencing at some loci

**Figure 8:**
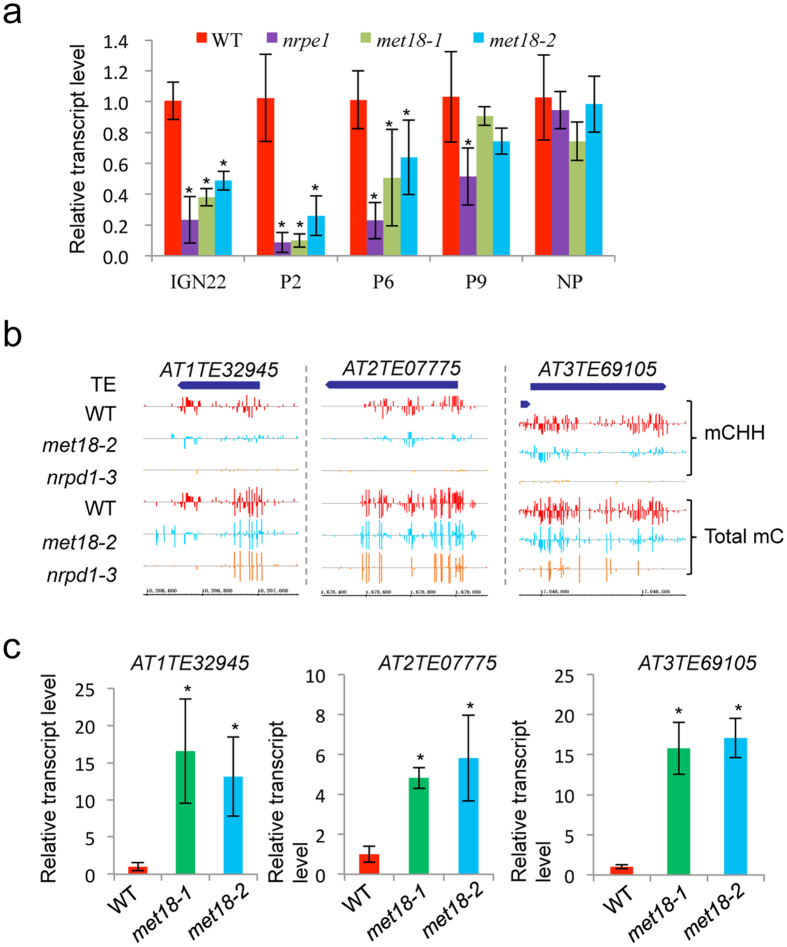
MET18 is required for the generation of Pol V-dependent noncoding RNAs and transcriptional gene silencing. (**a**) Detection of Pol V-dependent noncoding RNAs by real-time PCR, NP is a none NRPE1 enrichment region. (**b**,**c**) Snapshots in the Integrated Genome Browser showing the DNA methylation levels, and bar diagrams showing the real-time PCR-based expression analysis of the hypomethylated TEs in different genotypes. *TUB8* serves as an internal control. Standard errors were calculated from three biological replications, *P < 0.05.

In parallel with the whole-genome bisulfite sequencing, we characterized 24-nt siRNAs in *met18-2*, *nrpd1-3* and *nrpe1-11*. The total amount of 24-nt siRNAs in *met18-2* was reduced but the reduction was less compared to that in *nrpd1-3* and *nrpe1-11* (Supplementary Fig. 9a). Consistently, we identified 501, 4498 and 2963 siRNA-generating regions where 24-nt siRNAs were strongly decreased in *met18-2, nrpd1* and *nrpe1* respectively (Supplementary Table 6). Almost all (99.8%) MET18-regulated siRNAs are Pol IV-dependent (Supplementary Fig. 9b), in agreement with the fact that Pol IV is required for the production of the majority of 24-nt siRNAs. Pol V is also required for the production of most MET18-dependent siRNAs, although the proportion of siRNAs affected by Pol V is smaller (80.6%) (Supplementary Fig. 9b). We further examined whether the generation of Pol V transcripts was affected by MET18 dysfunction. Mutation of *MET18* reduced the levels of Pol V-dependent transcripts at multiple tested loci (Fig. 8a), including IGN22, P2 and P6^50^,^51^. The changes in 24-nt siRNA and Pol V transcript levels may lead to altered CHH DNA methylation level. To examine this, we classified siRNA-generating loci into four groups based on the dependence of siRNA generation on specific proteins and made detailed analysis. As expected, the slight reduction of CHH DNA methylation level in all groups coincided with the changes in 24-nt siRNA levels (Supplementary Fig. 9c). Altogether, our results suggest that MET18 was required for the accumulation of 24-nt siRNAs, Pol V transcripts and CHH methylation at some loci in *Arabidopsis*.

CHH methylation has been suggested to be critical for TE silencing. To determine whether CHH hypomethylation affects the expression of targeted TEs in *met18*, we examined the expression levels of three TEs which showed CHH DNA hypomethylation in *met18-2* (Fig. 8b). All of the tested TEs showed a substantial increase in their transcript levels (Fig. 8c). Recently, Han *et al.* reported that MMS19/MET18 associates with ABO4 and ICU2 to confer transcriptional gene silencing via a DNA methylation-independent mechanism^47^. To explore the genome-wide correlation between DNA hypo-methylation and expression of TEs or genes in *met18-2*, we analyzed our whole-genome bisulfite sequencing results and their RNAseq data and we identified 16 TEs showing increased expression from the CHH hypo-DMR list (Supplemental Table 7). Those 3 TEs showing increased expression as determined by our q-PCR experiments were not detected by the RNAseq assay possibly due to the low sensitivity of RNAseq (Fig. 8b,c). Thus, there might be more TEs whose expression is controlled by DNA methylation and MET18 may regulate TE expression through promoting DNA methylation. In contrast to TEs, none of the genes showing increased expression in *met18-2* as determined by RNAseq is hypomethylated, suggesting that MET18 regulates gene expression mainly through DNA methylation-independent mechanisms. Taken together, our results suggest that MET18 regulates transcriptional silencing through both DNA methylation-dependent and -independent mechanisms and the choice of mechanism is locus-specific in *Arabidopsis*.

## Discussion

In plants, active DNA demethylation is initiated by the ROS1/DME family of 5mC DNA glycosylases/lyases, and it is presumably continued through a base excision repair (BER) pathway^3^,^17^. Currently, little is known about how the enzymatic activity of ROS1 is regulated. To identify new components in the active DNA demethylation pathway initiated by ROS1, we designed a new chop-PCR marker based on the whole-genome DNA methylation analysis of *ros1-4*. We performed forward genetic screening for DNA hypermethylation mutants at the *EPF2* promoter region and identified MET18 as a new factor modulating DNA methylation level through regulating ROS1 enzymatic activity.

The effects of *MET18* mutations on DNA methylation and gene expression resembled those of the *ROS1* mutations, suggesting that MET18 and ROS1 may function in the same genetic pathway to prevent DNA hypermethylation. ROS1 and DME were co-purified with MET18 (Supplementary Table 5), and a Y-2-H assay as well as split-LUC assay demonstrated interaction between MET18 and ROS1 (Fig. 4). Western blot analysis results showed that MET18 was largely distributed in the cytoplasm, while ROS1 was mainly localized in the nucleus (Fig. 5c). It thus seems that MET18 and ROS1 are not present in the same cellular compartment. It is likely that MET18 regulates the incorporation of the Fe-S cluster into the ROS1 protein in the cytoplasm before ROS1 enters the nucleus. Our BiFC results confirmed their interaction in the cytoplasm and implicated the cytoplasmic regulation of ROS1 by MET18 (Fig. 4c). In *Arabidopsis*, the CIA pathway components NBP35, NAR1, CIA1, and CIA2/AE7 were reported to be essential for embryo development, and the null mutants for these components were lethal^45^,^52^. MET18 is not an essential component of the CIA complex in *Arabidopsis* as the *met18* knock-out mutants are viable in *Arabidopsis* and they did not show severe developmental phenotype under normal growth conditions^47^. It may act to enhance Fe-S cluster assembly for a small subset of Fe-S proteins, including the ROS1 family, but it does not mediate the assembly of the majority of the Fe-S proteins essential for plant development. Even for the ROS1 family, *MET18* mutation may only affect the efficiency of Fe-S cluster assembly, rather than totally abolishing the Fe-S cluster assembly, since the ROS1 activity was reduced but not totally abolished in *met18* (Fig. 5e,f). Moreover, not all of the ROS1 targets were affected in the *met18-2* mutant (Fig. 2). Another CIA pathway component AE7 has also been reported to mediate active DNA demethylation, and the DNA methylation levels in the *ae7-1* mutant were reported to be elevated at two loci (*XBAT34* and *MRD1*)^45^. However, in our study, the interaction between AE7 and ROS1 was not detected by either the Y-2-H assay or the split-LUC assay (Fig. 4), which was not consistent with the result got by Duan *et al.*^53^. Our whole-genome bisulfite sequencing results showed that the DNA methylation levels at the *XBAT34* and *MRD1* loci were also increased in *met18-2* (Supplementary Fig. 10). These results suggest that, although AE7 may not directly bind ROS1, both AE7 and MET18 are required for Fe-S cluster assembly into ROS1 protein.

As a key component of the CIA pathway, MET18/MMS19 was reported to be essential for the stability of the targeted proteins in human cells^38^. In contrast, our results showed that MET18 dysfunction in *Arabidopsis* had no effect on ROS1-GFP transgene stability and sub-localization although we could not detect the endogenous ROS1 protein due to the unavailability of an appropriate antibody. However, the mRNA level for ROS1 was found to be higher in *met18*, which is consistent with the findings in the *ros3* mutant^18^. Based on the ROS1 site-directed mutagenesis results and the previous finding regarding DME^46^, we propose that MET18 facilitates interaction between the Fe-S cluster assembly complex and the ROS1/DME family, and that it acts late in the Fe-S cluster assembly pathway (Supplementary Fig. 11). Dysfunction of MET18 therefore affects the enzymatic activity of ROS1.

MET18 may also regulate active DNA demethylation through facilitating Fe-S cluster assembly on other enzyme(s) involved in the BER pathway. In fission yeast, the replicative DNA polymerases have been demonstrated to be Fe-S proteins^54^. In *Arabidopsis*, Han *et al.* identified the catalytic subunits of DNA polymerases α, δ and ε (ICU2, AT5G63960, and ABO4) co-purified with MET18^47^. Consistently, we identified DNA polymerases subunits (AT5G63960 and ICU2) associated with MET18 (Supplementary Table 5). These results suggest that DNA polymerases in plants are very likely to be Fe-S proteins. Dysfunction of MET18 may thus compromise DNA polymerase activities and result in defective DNA demethylation. However, the identities of DNA polymerases that participate in the DNA demethylation pathway and whether their full activities require MET18 need further investigation. Besides DNA polymerases, other proteins involved in active DNA demethylation may also be Fe-S proteins and regulated by MET18 even though they do not have conserved Fe-S binding motifs.

When preparing our manuscript, Duan *et al.* reported that MET18 was required for preventing transgene silencing as well as active DNA demethylation through a different forward genetic screening^53^. The identification of the same protein by two independent studies further underlines the importance of MET18 in active DNA demethylation. More importantly, in our study, we investigated the precise role of MET18 in active DNA demethylation and found that the ROS1 enzymatic activity was compromised in *met18-2* (Fig. 5e,f). In addition to the DNA hypermethylation phenotype, we also identified lots of hypo-DMRs, especially in the CHH context in *met18-2*. Most of the CHH hypo-DMRs were also identified in *nrpd1-3* and *nrpe1-11*. Dysfunction of MET18 resulted in reduced production of Pol IV-dependent 24-nt siRNAs and Pol V-dependent scaffold transcripts and eventually led to abolished transcriptional gene silencing (Fig. 8), suggested that MET18 plays a role in modulating the RdDM pathway. In fission yeast, during DNA replication, Mms19 interacts with Cdc20 and Dos2 to recruit Pol II to produce siRNAs, which mediate heterochromatin silencing^40^. In *Arabidopsis,* however, whether MET18 modulates the RdDM pathway through similar mechanisms or through the CIA pathway remains unknown.

In summary, our study reveals that *MET18* mutation caused DNA hypermethylation at more than 1000 loci, of which about 70% were overlapped with the ROS1 targeted loci. This indicates that MET18 and ROS1 belong to the same genetic pathway. Our results clearly indicated that MET18 regulates active DNA demethylation by affecting the enzymatic activity of the ROS1/DME family proteins. Meanwhile, MET18 dysfunction gave rise to a DNA hypomethylation phenotype partially similar to that in RdDM mutants. Thus, MET18 can regulate both active DNA demethylation and DNA methylation pathways in *Arabidopsis*.

## Methods

### Plant materials and growth conditions

The *met18-1* (SALK_121963) and *met18-2* (SALK_147068) seeds were kindly provided by Dr. Xiaofeng Cui. *Arabidopsis* seedlings (Columbia background) were grown on Murashige-Skoog (MS) nutrient agar plates at 23 °C with 16 h of light and 8 h of darkness. After two weeks, the seedlings were transplanted into soil and grown at 23 °C with a 16 h photoperiod. *Nicotiana benthamiana* were also grown under the similar conditions.

### Chop-PCR analysis

A number of loci with higher DNA methylation levels than those of the WT Col-0 plants were identified on the basis of the genome-wide DNA methylation profiling of *ros1-4*. Based on the DNA methylation data, PCR-based markers were developed for detecting DNA methylation status^20^. For the chop-PCR analysis (detection of DNA methylation levels by PCR after DNA digestion by methylation-sensitive restriction enzymes), 1 μg of genomic DNA was digested with a methylation-sensitive enzyme (*HhaI, BstUI* or *HpaII*) in a 20 μl reaction mixture. PCR was performed using 1 μl of the digested DNA as template in a 10 μl reaction mixture using the loci-specific primers (Supplementary Table 8).

### Cloning strategy

For the complementation studies, an 8.5 kb genomic DNA fragment of the *MET18* gene was amplified from Col-0 by PCR and then cloned into the pCAMBIA1305 vector using an In-Fusion® HD Cloning Kit (Clontech). Full-length *ROS1* CDS was amplified by PCR and introduced into sp1300:GFP (or Flag tag) with *Sal*I and *Spe*I digestion. A *ROS1* mutation site was introduced into *Psuper::ROS1:3Flag* (super1300 vector) through site-directed mutagenesis using a MutanBEST Kit according to the manufacturer’s instructions (TaKaRa). *Agrobacterium tumefacines* strains (GV3101) harboring different constructs were used for *Arabidopsis* transformation following the standard floral dipping method. Primary transformants were selected on an MS medium containing 50 mg L^−1^ hygromycin.

### Bisulfite sequencing

About 100 ng of genomic DNA was modified using the BisulFlash DNA Modification Kit (Epigentek) according to the manufacturer’s instructions. An aliquot (1 μl) of bisulfite-treated DNA was used for PCR in a reaction volume of 20 μl using ExTaq DNA polymerase (Takara) and gene-specific primers. The PCR amplified products were cloned into a pGEM-T Easy vector (Promega) and at least 20 independent clones of each sample were sequenced.

### Whole-genome bisulfite sequencing and data analysis

Genomic DNA was extracted from 2 g leaf tissues of 14-day-old seedlings using DNeasy Plant Mini Kit (Qiagen) and then sent for bisulfite treatment, library preparation and sequencing by the High-throughput Sequencing Center (Biodynamic Optical Imaging Center, Peking University). The raw paired end reads were trimmed and quality controlled by SeqPrep (https://github.com/jstjohn/SeqPrep) and Sickle (https://github.com/najoshi/sickle) with default parameters. All clean reads were mapped to the *Arabidopsis thaliana* TAIR 10 (10th release of the Arabidopsis thaliana genome sequence from the Arabidopsis Information Resource) genome with the BSMAP aligner allowing up to 2 mismatches. Uniquely mapped reads were used to determine the cytosine methylation levels as previously stated^4^. To identify DMRs, only cytosines covered with at least four reads in a library were considered. DMRs were searched by using a 200 bp sliding-window with 50 bp as step-size. DNA methylation levels of different plants were compared pairwise through Fisher’s exact test and the *p* values were adjusted for multiple comparisons using the Benjamini-Hochberg method. Windows with an adjusted *p* value less than 0.01 and an over 2-fold change in the methylation level were retained for further analysis. Moreover, the *p* value of each cytosine in the selected regions was calculated by Fisher’s exact test and it was considered as differentially methylated cytosine (DMC) if its *p* value was <0.01. The regions were retained only if it contained at least 7 DMCs. Finally neighboring DMRs were combined if the gap was <100 bp. The length of the DMR was adjusted to start from the first 5mC and end at the last 5mC.

### Small RNA Sequencing and data analysis

Total RNAs were extracted from 12-day-old seedlings using TRIzol reagent. Small RNA (sRNA) data were analyzed as described by Zhang *et al.*^55^.

### Yeast two-hybrid assay

For the yeast two-hybrid assays, coding sequences of *MET18, ROS1* and *AE7* were amplified by PCR. After verification of the sequences, the genes were cloned into pGBK-T7 or pGAD-T7 vector (Clontech) to generate DNA binding or activation domain fusion constructs, respectively. *Nde*I and *Bam*HI sites were used for cloning *MET18* and *AE7* in pGBKT7 and pGAD-T7, while *Nde*I and *Eco*RI sites were used for cloning *ROS1* in pGBKT7 and pGAD-T7. For the protein interaction analysis, two combinatory constructs were simultaneously transformed into yeast strain AH109 (Clontech) and then tested for Leu-, Trp- and His- auxotrophy according to the manufacturer’s protocols.

### Split-LUC assay

For the split-LUC assays, the coding sequences of *MET18* and *AE7* were amplified by PCR. After verification of the sequences, the genes were cloned into pCAMBIA1300-NLUC or pCAMBIA1300-CLUC to generate N terminal or C terminal luciferase protein fusion constructs, respectively. The *Kpn*I and *Bam*HI sites were used for cloning *MET18* and *AE7* in pCAMBIA1300-nLUC or pCAMBIA1300-cLUC. WT or mutant forms of ROS1 were introduced to nLUC using In-Fusion® HD Cloning Kit (Clontech). For the protein interaction analysis, two combinatory constructs were simultaneously transformed into *Nicotiana benthamiana* leaves. To prevent the silencing of the genes, a construct expressing viral p19 protein was co-transformed^56^ .

### Bimolecular fluorescence complementation (BiFC) assay

The BiFC assay was performed as described by Walter *et al.*^57^.

### Scaffold RNA and mRNA analyses

Total RNA was extracted from 14-day-old *Arabidopsis* seedlings using an RNeasy Plant Mini Kit (Qiagen), and any contaminating DNA was removed by RNase-free DNase (Qiagen) treatment. mRNA (2 μg) was utilized for the first-strand cDNA synthesis using the SuperScript^TM^ III First-Strand Synthesis System (Invitrogen) according to the manufacturer’s instructions. Oligo dT primers were used during RT reaction to analyze mRNA expression, while gene-specific primers were used for detecting Pol V-dependent noncoding RNAs. The cDNA was then diluted five times, and 2 μl was used as a template in a 25 μl reaction mixture using iQ SYBR Green Supermix (Bio-Rad). The primers used for real-time PCR are listed in Supplementary Table 8.

### Nuclear-cytoplasmic fractionation and Western blot analysis

Nuclear-cytoplasmic fractionation was performed as described by Wang *et al.*^58^. Briefly, 14-day-old MET18-3Flag transgenic plants (about 1 g) were ground in liquid N_2_ using a pestle and mortar. The resultant fine powder was suspended in 2 ml of lysis buffer (20 mM Tris-HCl, pH 7.5, 20 mM KCl, 2 mM EDTA, 2.5 mM MgCl_2_, 25% glycerol, 250 mM sucrose, 5 mM DTT and 1 protease inhibitor cocktail tablet (Roche)) and then centrifuged for 10 min at 1500 g at 4 °C after filtering through a double layer of Miracloth. The supernatant was centrifuged for 10 min at 10,000 g at 4 °C and the cytoplasmic fraction (supernatant) was collected. The pellet (nuclear fraction) was washed four times with nuclear resuspension buffer NRBT (20 mM Tris-HCl, pH 7.4, 25% glycerol, 2.5 mM MgCl_2_, and 0.2% Triton X-100) and then resuspended in NRB2 buffer (20 mM Tris-HCl, pH 7.5, 0.25 M sucrose, 10 mM MgCl_2_, 0.5% Triton X-100, and 5 mM DTT) before being overlaid on top of NRB3 buffer (20 mM Tris-HCl, pH 7.5, 1.7 M sucrose, 10 mM MgCl_2_, 0.5% Triton X-100, and 5 mM DTT) and centrifuged for 45 min at 16,000 g at 4 °C. HSP90 was detected using a rabbit anti-HSP90 polyclonal antibody (1:5,000, at-115; Santa Cruz Biotechnology) as the cytoplasmic marker, and histone H3 (05-499 Millipore) was used as the nuclear marker. MET18-3Flag was detected by anti-Flag (Sigma, F1804).

### Fluorescence microscopy

ROS1-GFP fluorescence was detected with a confocal microscope (Zeiss, LSM710).

### ROS1 *in vitro* activity assay

The ROS1 *in vitro* activity assay was performed as described by Mok *et al.*^46^. Briefly, WT control and two mutant forms (C1038S and C1045S) of full-length ROS1 CDS were cloned into pMal-c2X vector (New England Biolabs). The pMal-c2X, pMal-WT-ROS1, pMal-C1038S and pMal-C1045S were transformed into the *E. coli* strain DH5α. Overnight cultures of the four above-mentioned four transformed bacteria were diluted to 1.0 O.D. and then grown on LB agar medium containing 100 mg L^−1^ ampicillin with or without 20 μM IPTG at 37 °C for 16 h.

To compare the ROS1 activity in WT and *met18* mutant plants, approximately 5 g of floral tissues from *pSP1300::ROS1-GFP* transgenic plants in *ros1-4* or *met18-2* background were grounded into a fine powder using liquid N_2_ and then suspended in 25 ml lysis buffer (50 mM Tris pH8.0, 230 mM NaCl, 5 mM MgCl_2_, 10% glycerol, 0.2% NP-40, 0.5 mM DTT, 1 mM PMSF and 1 protease inhibitor cocktail tablet (Roche) and then centrifuged for 15 min at 18300 g 4 °C. The supernatants was incubated with 12.5 μl anti-GFP tag rabbit polyclonal antibody (EAZYBIO) at 4 °C for 2 h. About 50 μl of Protein A agarose (Chromo Tek) beads were added to trap the anti-GFP antibody and incubated at 4 °C for another 2 h. The beads were washed twice with 25 ml lysis buffer and twice with 1 ml lysis buffer.

The nicking activity assay was performed as described by Agius *et al.*^13^. Plasmid pBluescript KS+ (Stratagene) was purified from *E. coli* Trans110 (TRANSGEN BIOTECH), a *dam* and *dcm* strain. About 50 μg of the plasmid was methylated *in vitro* in a 50 μl reaction containing 5 U of SssI methylase (New England Biolabs) under the conditions recommended by the manufacturer. The non-methylated plasmid was subjected to the same procedure, but without the addition of the methyltransferase. For the nicking activity assay, a 20 μl reaction mixture containing 400 ng of each of the plasmid and 10 mM Tris-HCl, pH 7.9, 50 mM NaCl, 10 mM MgCl_2_, 1 mM DTT, 0.2 mg ml^−1^ BSA was incubated overnight at 37 °C with an equal amount of ROS1-GFP protein from either *ros1-4* or *met18-2* background.

### Affinity purification and mass spectrometry

About 6 g of flower tissue collected from MET18-3Falg transgenic plants was ground into fine powder in liquid N_2_ using a pestle and mortar and then suspended in 25 ml of lysis buffer (50 mM Tris, pH8.0, 230 mM NaCl, 5 mM MgCl_2_, 10% glycerol, 0.2% NP-40, 0.5 mM DTT, 1 mM PMSF, and 1 protease inhibitor cocktail tablet (Roche)) and centrifuged for 20 min at 18,300 g at 4 °C. The supernatant was incubated with 50 μl of anti-Flag M2 Magnetic beads (Sigma) for 3 h at 4 °C. The beads were washed twice with 25 ml of lysis buffer and then four times with 1 ml of lysis buffer. Proteins bound to the Flag beads were eluted with Flag peptides (Sigma). After SDS-PAGE, the entire gel lane was excised into six equal portions and digested in-gel with sequencing grade trypsin, followed by the mass spectrometric analysis as previously described by Li *et al.*^25^.

## Additional Information

**Accession codes:** Whole-genome bisulfite sequencing was used to analyze the genome-wide methylation status in WT (Col-0) and *met18-2* mutant plants. The dataset was deposited at NCBI (Col-0: SRX747290, *met18-2*: SRX747291). The *ros1-4* and *rdd* whole-genome bisulfite sequencing data used were from GEO accession GSE33071^20^ and *nrpd1-3* and *nrpe1-11* methylation data were from GSE44209^55^. The small RNA dataset was deposited at NCBI (PRJNA299084).

**How to cite this article**: Wang, X. *et al.* The cytosolic Fe-S cluster assembly component MET18 is required for the full enzymatic activity of ROS1 in active DNA demethylation. *Sci. Rep.* **6**, 26443; doi: 10.1038/srep26443 (2016).

## Supplementary Material

Supplementary Information

Supplementary Table 1

Supplementary Table 2

Supplementary Table 3

Supplementary Table 4

Supplementary Table 5

Supplementary Table 6

Supplementary Table 7

## References

[b1] Law J. A. & Jacobsen S. E. Establishing, maintaining and modifying DNA methylation patterns in plants and animals. Nat Rev Genet 11, 204–220 (2010). 20142834 10.1038/nrg2719 PMC3034103

[b2] Zilberman D. The evolving functions of DNA methylation. Curr Opin Plant Biol 11, 554–559 (2008). 18774331 10.1016/j.pbi.2008.07.004

[b3] Zhu J. K. Active DNA demethylation mediated by DNA glycosylases. Annu Rev Genet 43, 143–166 (2009). 19659441 10.1146/annurev-genet-102108-134205 PMC3137514

[b4] Lister R. *et al.* Highly integrated single-base resolution maps of the epigenome in *Arabidopsis*. Cell 133, 523–536 (2008). 18423832 10.1016/j.cell.2008.03.029 PMC2723732

[b5] Zhang X. *et al.* Genome-wide high-resolution mapping and functional analysis of DNA methylation in *Arabidopsis*. Cell 126, 1189–1201 (2006). 16949657 10.1016/j.cell.2006.08.003

[b6] Zhang H. & Zhu J. K. Active DNA demethylation in plants and animals. Cold Spring Harb Symp Quant Biol 77, 161–173 (2012). 23197304 10.1101/sqb.2012.77.014936 PMC3657592

[b7] Zemach A. *et al.* The *Arabidopsis* nucleosome remodeler DDM1 allows DNA methyltransferases to access H1-containing heterochromatin. Cell 153, 193–205 (2013). 23540698 10.1016/j.cell.2013.02.033 PMC4035305

[b8] Wu S. C. & Zhang Y. Active DNA demethylation: many roads lead to Rome. Nat Rev Mol Cell Biol 11, 607–620 (2010). 20683471 10.1038/nrm2950 PMC3711520

[b9] Gong Z. *et al.* *ROS1*, a repressor of transcriptional gene silencing in *Arabidopsis*, encodes a DNA glycosylase/lyase. Cell 111, 803–814 (2002). 12526807 10.1016/s0092-8674(02)01133-9

[b10] Ortega-Galisteo A. P., Morales-Ruiz T., Ariza R. R. & Roldán-Arjona T. *Arabidopsis* DEMETER-LIKE proteins DML2 and DML3 are required for appropriate distribution of DNA methylation marks. Plant Mol Biol 67, 671–681 (2008). 18493721 10.1007/s11103-008-9346-0

[b11] Choi Y. *et al.* DEMETER, a DNA glycosylase domain protein, is required for endosperm gene imprinting and seed viability in *Arabidopsis*. Cell 110, 33–42 (2002). 12150995 10.1016/s0092-8674(02)00807-3

[b12] Morales-Ruiz T. *et al.* *DEMETER* and *REPRESSOR OF SILENCING 1* encode 5-methylcytosine DNA glycosylases. Proc Natl Acad Sci USA 103, 6853–6858 (2006). 16624880 10.1073/pnas.0601109103 PMC1458983

[b13] Agius F., Kapoor A. & Zhu J. K. Role of the *Arabidopsis* DNA glycosylase/lyase ROS1 in active DNA demethylation. Proc Natl Acad Sci USA 103, 11796–11801 (2006). 16864782 10.1073/pnas.0603563103 PMC1544249

[b14] Gehring M. *et al.* DEMETER DNA glycosylase establishes MEDEA polycomb gene self-imprinting by allele-specific demethylation. Cell 124, 495–506 (2006). 16469697 10.1016/j.cell.2005.12.034 PMC4106368

[b15] Martínez-Macías M. I. *et al.* A DNA 3′ phosphatase functions in active DNA demethylation in *Arabidopsis*. Mol Cell 45, 357–370 (2012). 22325353 10.1016/j.molcel.2011.11.034 PMC3278721

[b16] Li Y. *et al.* An AP endonuclease functions in active DNA dimethylation and gene imprinting in *Arabidopsis*. PLoS Genet 11, e1004905 (2015). 25569774 10.1371/journal.pgen.1004905 PMC4287435

[b17] Gong Z. & Zhu J. K. Active DNA demethylation by oxidation and repair. Cell Res 21, 1649–1651 (2011). 21862972 10.1038/cr.2011.140 PMC3357985

[b18] Zheng X. *et al.* ROS3 is an RNA-binding protein required for DNA demethylation in *Arabidopsis*. Nature 455, 1259–1262 (2008). 18815596 10.1038/nature07305 PMC2782394

[b19] Stroud H., Greenberg M. V., Feng S., Bernatavichute Y. V. & Jacobsen S. E. Comprehensive analysis of silencing mutants reveals complex regulation of the *Arabidopsis* methylome. Cell 152, 352–364 (2013). 23313553 10.1016/j.cell.2012.10.054 PMC3597350

[b20] Qian W. *et al.* A histone acetyltransferase regulates active DNA demethylation in *Arabidopsis*. Science 336, 1445–1448 (2012). 22700931 10.1126/science.1219416 PMC3575687

[b21] Qian W. *et al.* Regulation of active DNA demethylation by an α-crystallin domain protein in *Arabidopsis*. Mol Cell 55, 361–371 (2014). 25002145 10.1016/j.molcel.2014.06.008 PMC4302764

[b22] Lang Z. *et al.* The Methyl-CpG-Binding Protein MBD7 facilitates active DNA demethylation to limit DNA hyper-methylation and transcriptional gene silencing. Mol Cell 57, 971–983 (2015). 25684209 10.1016/j.molcel.2015.01.009 PMC4369450

[b23] Wang C. *et al.* Methyl-CpG-Binding Domain Protein MBD7 is required for active DNA demethylation in *Arabidopsis*. Plant Physiol 167, 905–914 (2015). 25593350 10.1104/pp.114.252106 PMC4348759

[b24] Li X. *et al.* Antisilencing role of the RNA-directed DNA methylation pathway and a histone acetyltransferase in *Arabidopsis*. Proc Natl Acad Sci USA 109, 11425–11430 (2012). 22733760 10.1073/pnas.1208557109 PMC3396497

[b25] Li Q. *et al.* Regulation of Active DNA Demethylation by a Methyl-CpG-Binding Domain Protein in *Arabidopsis thaliana*. PLoS Genet 11, e1005210 (2015). 25933434 10.1371/journal.pgen.1005210 PMC4416881

[b26] Zhao Y. *et al.* *REPRESSOR OF SILENCING5* encodes a member of the small heat shock protein family and is required for DNA demethylation in *Arabidopsis*. Plant Cell 26, 2660–2675 (2014). 24920332 10.1105/tpc.114.126730 PMC4114958

[b27] Buzas D. M., Nakamura M. & Kinoshita T. Epigenetic role for the conserved Fe-S cluster biogenesis protein AtDRE2 in *Arabidopsis thaliana*. Proc Natl Acad Sci USA 111, 13565–13570 (2014). 25197096 10.1073/pnas.1404058111 PMC4169955

[b28] Nakamura M. *et al.* The role of *Arabidopsis thaliana* NAR1, a cytosolic iron-sulfur cluster assembly component, in gametophytic gene expression and oxidative stress responses in vegetative tissue. New Phytol 199, 925–935 (2013). 23734982 10.1111/nph.12350

[b29] Balk J. & Pilon M. Ancient and essential: the assembly of iron-sulfur clusters in plants. Trends Plant Sci 16, 218–226 (2011). 21257336 10.1016/j.tplants.2010.12.006

[b30] Netz D. J., Mascarenhas J., Stehling O., Pierik A. J. & Lill R. Maturation of cytosolic and nuclear iron-sulfur proteins. Trends Cell Biol 24, 303–312 (2014). 24314740 10.1016/j.tcb.2013.11.005

[b31] Lill R. & Mühlenhoff U. Iron-sulfur protein biogenesis in eukaryotes: components and mechanisms. Annu Rev Cell Dev Biol 22, 457–486 (2006). 16824008 10.1146/annurev.cellbio.22.010305.104538

[b32] Lill R. & Mühlenhoff U. Maturation of iron-sulfur proteins in eukaryotes: mechanisms, connected processes, and diseases. Annu Rev Biochem 77, 669–700 (2008). 18366324 10.1146/annurev.biochem.76.052705.162653

[b33] Kispal G., Csere P., Prohl C. & Lill R. The mitochondrial proteins Atm1p and Nfs1p are essential for biogenesis of cytosolic Fe/S proteins. EMBO J 18, 3981–3989 (1999). 10406803 10.1093/emboj/18.14.3981 PMC1171474

[b34] Adam A. C., Bornhövd C., Prokisch H., Neupert W. & Hell K. The Nfs1 interacting protein Isd11 has an essential role in Fe/S cluster biogenesis in mitochondria. EMBO J 25, 174–183 (2006). 16341090 10.1038/sj.emboj.7600905 PMC1356348

[b35] Bych K. *et al.* The essential cytosolic iron-sulfur protein Nbp35 acts without Cfd1 partner in the green lineage. J Biol Chem 283, 35797–35804 (2008). 18957412 10.1074/jbc.M807303200

[b36] Balk J. & Lobréaux S. Biogenesis of iron-sulfur proteins in plants. Trends Plant Sci 10, 324–331 (2005). 15951221 10.1016/j.tplants.2005.05.002

[b37] Tsaousis A. D., Gentekaki E., Eme L., Gaston D. & Roger A. J. Evolution of the cytosolic iron-sulfur cluster assembly machinery in *Blastocystis* species and other microbial eukaryotes. Eukaryot Cell 13, 143–153 (2014). 24243793 10.1128/EC.00158-13 PMC3910952

[b38] Stehling O. *et al.* MMS19 assembles iron-sulfur proteins required for DNA metabolism and genomic integrity. Science 337, 195–199 (2012). 22678362 10.1126/science.1219723 PMC3420340

[b39] Gari K. *et al.* MMS19 links cytoplasmic iron-sulfur cluster assembly to DNA metabolism. Science 337, 243–245 (2012). 22678361 10.1126/science.1219664

[b40] Li F., Martienssen R. & Cande W. Z. Coordination of DNA replication and histone modification by the Rik1-Dos2 complex. Nature 475, 244–248 (2011). 21725325 10.1038/nature10161 PMC3163161

[b41] Ito S. *et al.* MMXD, a TFIIH-independent XPD-MMS19 protein complex involved in chromosome segregation. Mol Cell 39, 632–640 (2010). 20797633 10.1016/j.molcel.2010.07.029

[b42] Kou H., Zhou Y., Gorospe R. M. & Wang Z. Mms19 protein functions in nucleotide excision repair by sustaining an adequate cellular concentration of the TFIIH component Rad3. Proc Natl Acad Sci USA 105, 15714–15719 (2008). 18836076 10.1073/pnas.0710736105 PMC2572961

[b43] Askree S. H. *et al.* A genome-wide screen for *Saccharomyces cerevisiae* deletion mutants that affect telomere length. Proc Natl Acad Sci USA 101, 8658–8663 (2004). 15161972 10.1073/pnas.0401263101 PMC423251

[b44] Lauder S. *et al.* Dual requirement for the yeast MMS19 gene in DNA repair and RNA polymerase II transcription. Mol Cell Biol 16, 6783–6793 (1996). 8943333 10.1128/mcb.16.12.6783 PMC231681

[b45] Luo D., Bernard D. G., Balk J., Hai H. & Cui X. The DUF59 family gene *AE7* acts in the cytosolic iron-sulfur cluster assembly pathway to maintain nuclear genome integrity in *Arabidopsis*. Plant Cell 24, 4135–4148 (2012). 23104832 10.1105/tpc.112.102608 PMC3517241

[b46] Mok Y. G. *et al.* Domain structure of the DEMETER 5-methylcytosine DNA glycosylase. Proc Natl Acad Sci USA 107, 19225–19230 (2010). 20974931 10.1073/pnas.1014348107 PMC2984145

[b47] Han Y. F. *et al.* The cytosolic iron-sulfur cluster assembly protein MMS19 regulates transcriptional gene silencing, DNA repair, and flowering time in *Arabidopsis*. PLoS One 10, e0129137 (2015). 26053632 10.1371/journal.pone.0129137 PMC4459967

[b48] Williams B. P., Pignatta D., Henikoff S. & Gehring M. Methylation-sensitive expression of a DNA demethylase gene serves as an epigenetic rheostat. PLoS Genet 11, e1005142 (2015). 25826366 10.1371/journal.pgen.1005142 PMC4380477

[b49] Lei M. *et al.* Regulatory link between DNA methylation and active demethylation in *Arabidopsis*. Proc Natl Acad Sci USA 112, 3553–3557 (2015). 25733903 10.1073/pnas.1502279112 PMC4371987

[b50] Zhong X. *et al.* DDR complex facilitates global association of RNA polymerase V to promoters and evolutionarily young transposons. Nat Struct Mol Biol 19, 870–875 (2012). 22864289 10.1038/nsmb.2354 PMC3443314

[b51] Wierzbicki A. T., Haag J. R. & Pikaard C. S. Noncoding transcription by RNA polymerase Pol IVb/Pol V mediates transcriptional silencing of overlapping and adjacent genes. Cell 135, 635–648 (2008). 19013275 10.1016/j.cell.2008.09.035 PMC2602798

[b52] Kohbushi H. *et al.* *Arabidopsis* cytosolic Nbp35 homodimer can assemble both [2Fe-2S] and [4Fe-4S] clusters in two distinct domains. Biochem Biophys Res Commun 378, 810–815 (2009). 19084504 10.1016/j.bbrc.2008.11.138

[b53] Duan C. G. *et al.* MET18 Connects the Cytosolic Iron-Sulfur Cluster Assembly Pathway to Active DNA Demethylation in *Arabidopsis*. PLoS Genet 11, e1005559 (2015). 26492035 10.1371/journal.pgen.1005559 PMC4619598

[b54] Netz D. J. *et al.* Eukaryotic DNA polymerases require an iron-sulfur cluster for the formation of active complexes. Nat Chem Biol 8, 125–132 (2012). 22119860 10.1038/nchembio.721 PMC3241888

[b55] Zhang H. *et al.* DTF1 is a core component of RNA-directed DNA methylation and may assist in the recruitment of Pol IV. Proc Natl Acad Sci USA 110, 8290–8295 (2013). 23637343 10.1073/pnas.1300585110 PMC3657815

[b56] Shamloul M., Trusa J., Mett V. & Yusibov V. Optimization and utilization of Agrobacterium-mediated transient protein production in Nicotiana. J Vis Exp 86, doi: 10.3791/51204 (2014). PMC4174718 24796351

[b57] Walter M. *et al.* Visualization of protein interactions in living plant cells using bimolecular fluorescence complementation. Plant J 40, 428–438 (2004). 15469500 10.1111/j.1365-313X.2004.02219.x

[b58] Wang W. *et al.* An importin β protein negatively regulates microRNA activity in *Arabidopsis*. Plant Cell 23, 3565–3576 (2011). 21984696 10.1105/tpc.111.091058 PMC3229135

