# Supplementary material for: The cytosolic Fe-S cluster assembly component MET18 is required for the full enzymatic activity of ROS1 in active DNA demethylation
Source: Sci Rep. 2016 May 19;6:26443. doi: 10.1038/srep26443 (PMC4872223; doi:10.1038/srep26443)
Supplement: Supplementary Information [file srep26443-s1.pdf]

Supplementary information

The cytosolic Fe-S cluster assembly component MET18 is required for the full enzymatic activity of ROS1 in active DNA demethylation

Xiaokang Wang[1,3], Qi Li[1,3], Wei Yuan[1], Zhendong Cao[1], Bei Qi[1], Suresh Kumar[2], Yan Li[1] and Weiqiang Qian[1*]

[1]State Key Laboratory of Protein and Plant Gene Research, The Peking-Tsinghua Center for Life Sciences, School of Advanced Agricultural Sciences and School of Life Sciences, Peking University, Beijing 100871, China

[2]Division of Biochemistry, Indian Agricultural Research Institute, New Delhi 110012, India

[3]These authors contributed equally to this work

Supplementary Table 1. List of T-DNA insertion mutant library.

Supplementary Table 2. List of hyper-DMRs in *met18-2, ros1-4* and *rdd*.

Supplementary Table 3. List of hypo-DMRs in the CHH context in *met18-2, nrpd1-3* and *nrpe1-11*.

Supplementary Table 4. List of the unique peptides identified from mass-spectrometric analysis.

Supplementary Table 5. List of the proteins co-purified with MET18.

Supplementary Table 6. List of the differentially expressed siRNA clusters identified in *nrpd1*, *nrpe1* and *met18-2.*

Supplementary Table 7. List of up-regulated TEs in *met18-2*.

Supplementary Table 8. Primers used in this study.

**Primer Name**	**Sequence (5'-3')**	**Purpose**
At1g26400-F1	TGACCTGCATAGGCTATAACACA	Chop-PCR
At1g26400-R1	ATTGGAATCAATCCGAGTGG	
At1g26390-F1	GAACCTGAGTGCTGAAGGTTACTC	
At1g26390-R1	CAAGATTCAATACTTTACGACATG	
EPF2-F1	AATATGATCGATGGGCTTCTTG	
EPF2-R1	AAAGAGGCACACATGTTGAAGG	
At1g26390-F2	GTTGTTTTGGGTAGATTATTTAGTTGGTATT	Bisulfite sequencing
At1g26390-R2	TCAATCTCTTCAAATTCCCCAAAAAATACTTAA	
At1g26400-F2	GTAGTTTGAGATGATTAATGATAGAGTT	
At1g26400-R2	AAACTTATTCAATCTTCAATACTCTAC	
SALK_121963 LP	CTTTGGAGAATGATTCTTTATC	Genotyping
SALK_121963 RP	CATAGCATAAACAAGTAGATCAC	
SALK_147068 LP	GAATTCCCAGACTTCACTATCGAG	
SALK_147068 RP	CATTAGATGCGGATATGATGTAAG	
MET18-CLUC-F	GGTACCATGATGGTAGAACCGAATCAGC	split-LUC
MET18-CLUC-R	GGATCCTCAGAATATGTTACTTCCGGATGT	
AE7-N/CLUC-F	GGTACCATGGTATCTGGGTTGATTAA	
AE7-NLUC-R	GTCGACCTCTTCTGATGGCAGGCATTC	
AE7-CLUC-R	GTCGACTCACTCTTCTGATGGCAGGC	
MET18-BD-F	CATATGTACTACCATCTTGGCTTAGT	
MET18-BD-R	GGATCCAGTCTTATACAATGAAGGCC	
AE7-AD/BD-F	CATATGATGGTATCTGGGTTGATTAA	
AE7-AD/BD-R	GGATCCTCACTCTTCTGATGGCAGGC	
ROS1-AD-F	CATATGGAGAAACAGAGGAGAGAAGAAAGC	

ROS1-AD-R	GAATTCTTAGGCGAGGTTAGCTTGTTGTC	
ROS1-C1045S-F<br>*ROS1*-C1045S-R	CCTAATTcCAATGCATGTCCG<br>TTTGCTCTTTGTGCAAAAGAC	Site directed mutagenesis
ROS1-SP1300-F<br>*ROS1*-SP1300-R	AGTCGTCGACATGGAGAAACAGAGGAGAGAAGAAAGC<br>AGTCACTAGTGGCGAGGTTAGCTTGTTGTCCCT	Overexpression of ROS1
EPF2-F2<br>*EPF2*-R2	CTCAGTCGCCGAATCATGTA<br>CGCCAGCCAAAACTGATATT	Real-time PCR
AT5G38550-F<br>*AT5G38550*-R	GCATATTATCGCCCGATTCC<br>CAGATGAAAGGGTCTTGTTGC	
AT5G38540-F<br>*AT5G38540*-R	AATCTGTTGACGCTTACTAC<br>CAATGATCTTCTTTCCTTTG	
AT1TE32945-F<br>*AT1TE32945-R*	TGGTGGACGGATGGTAGAGT<br>AAGCAGCGGCAGGAAGATTT	
AT2TE07775-F<br>*AT2TE07775-R*	AAATTCCCAAAACAACACGA<br>GCATAATACCCGAACCCTAA	
AT3TE69105-F<br>*AT3TE69105-R*	GCGTGAGGAAGGTGAAAATA<br>AAGCCATGGAGTGCATAAGA	
ROS1-F<br>*ROS1*-R	AAGGACCAACTTGTTGCGAC<br>AGGACTCTATTAGCACTGAGC	
LUC-F<br>*LUC*-R	AAGCGAAGGTTGTGGATCTGGA<br>TCAATCAAGGCGTTGGTCGCTT	
NPTII-F<br>*NPTII*-R	ACCTTGCTCCTGCCGAGAAAGTAT<br>ATGCGATCTTTCGCTTGGTGGT	
IGN22-F<br>IGN22-R	CGGGTCCTTGGACTCCTGAT<br>TCGTGACCGGAATAATTAAATGG	
P2-F<br>P2-R	CTAAAGCCCATCAGAGAAACC<br>GCTTTGATTGTTTTAACCGGTG	
P6-F<br>P6-R	GGCTTCGATAGGAAGAATGCCC<br>GTGAAACTGCCAGATCCAAATTC	
P9-F<br>P9-R	CCGTTTCTGGGTAGGTCGGC<br>CCAATTCTTGACTGGAGTGGAC	
NP-F<br>NP-R	GTTCAATGAATAAGAATCACTGAG<br>CCATGTCTTGTGCATTGTCAGAATCAG	

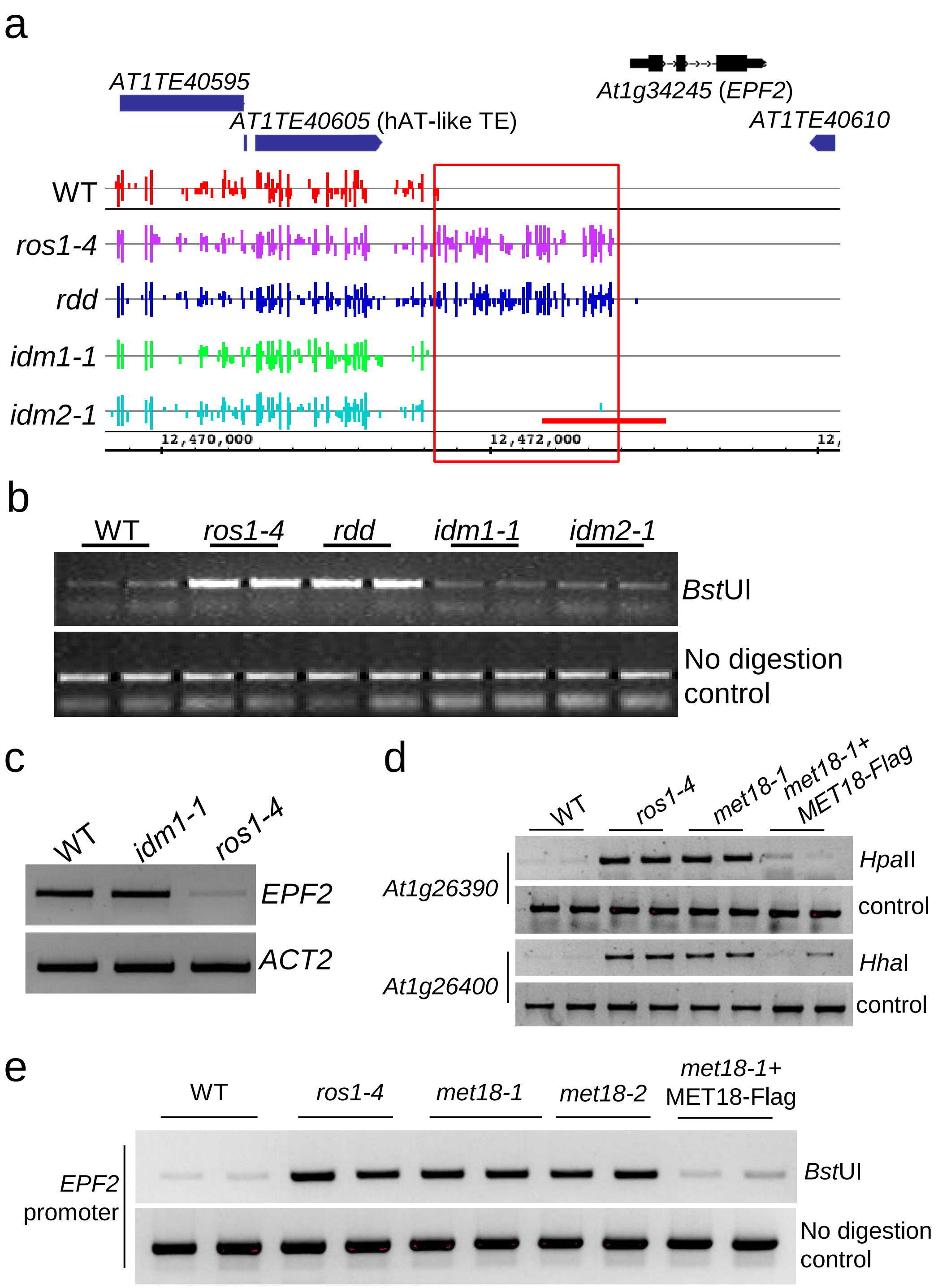

Supplementary Figure 1. The rationale of designing chop-PCR marker at the *EPF2* promoter region. (**a**) Snapshot in the Integrated Genome Browser showing the DNA methylation levels at the *EPF2* promoter and upstream region in the WT and mutant plants. The regions specifically hypermethylated in *ros1-4* and *rdd* are highlighted with red box. The chop-PCR marker region is highlighted with horizontal red line. (**b**) Analysis of the DNA methylation levels at the *EPF2* promoter by chop-PCR. Methylation-sensitive restriction enzyme *Bst*UI was used for digestion of genomic DNA. (**c**) RT-PCR analysis of *EPF2* transcript levels in *idm1-1* and *ros1-4*. *ACT2* serves as a control. (**d-e**) Complementation assay: chop-PCR detection of DNA methylation levels at *At1g26390* and *At1g26400* loci (**d**) and the *EPF2* promoter . (**e**) Methylation-sensitive restriction enzymes *Hpa*II, *Hha*I and *BstU*I were used for digestion of genomic DNA.

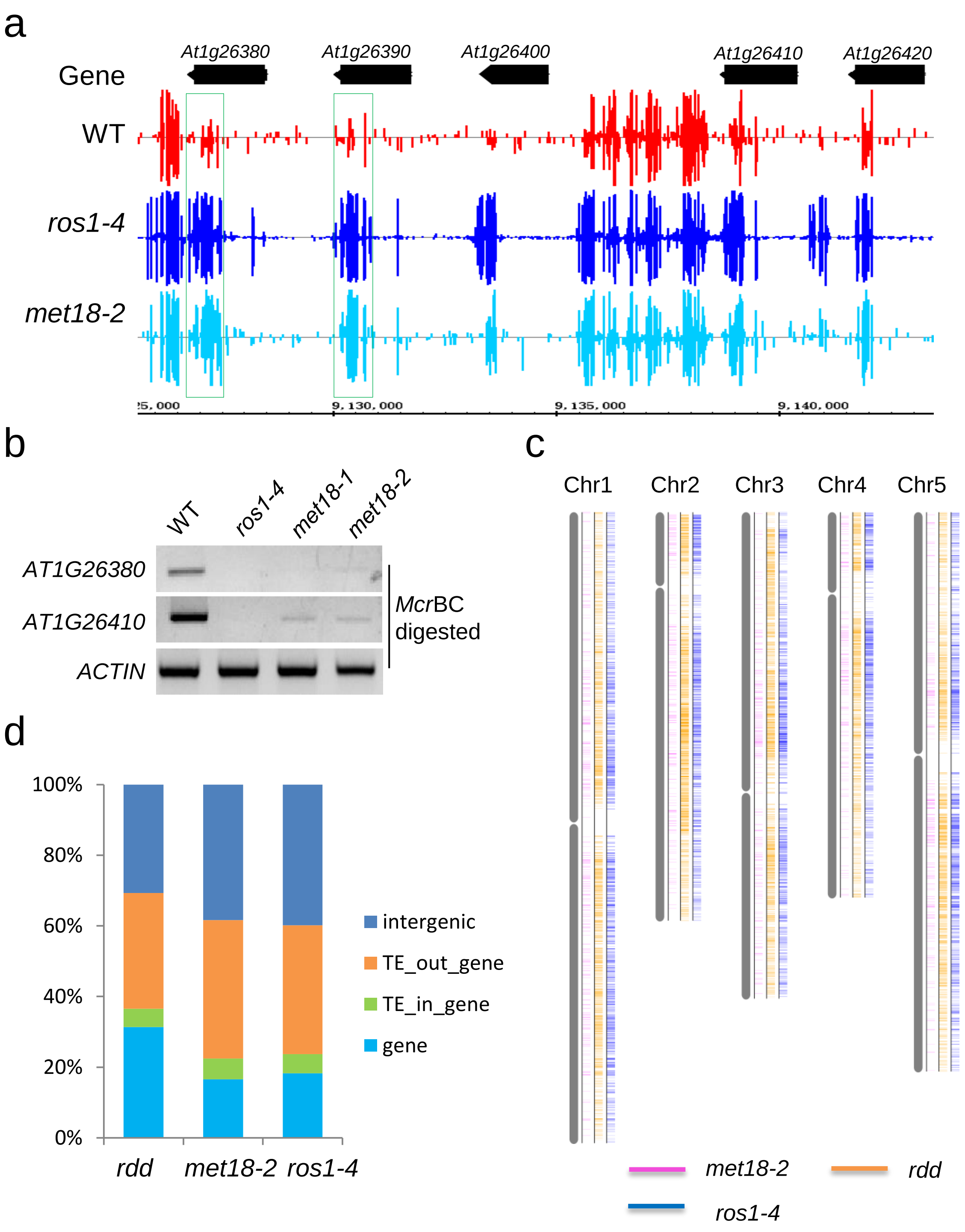

Supplementary Figure 2

e

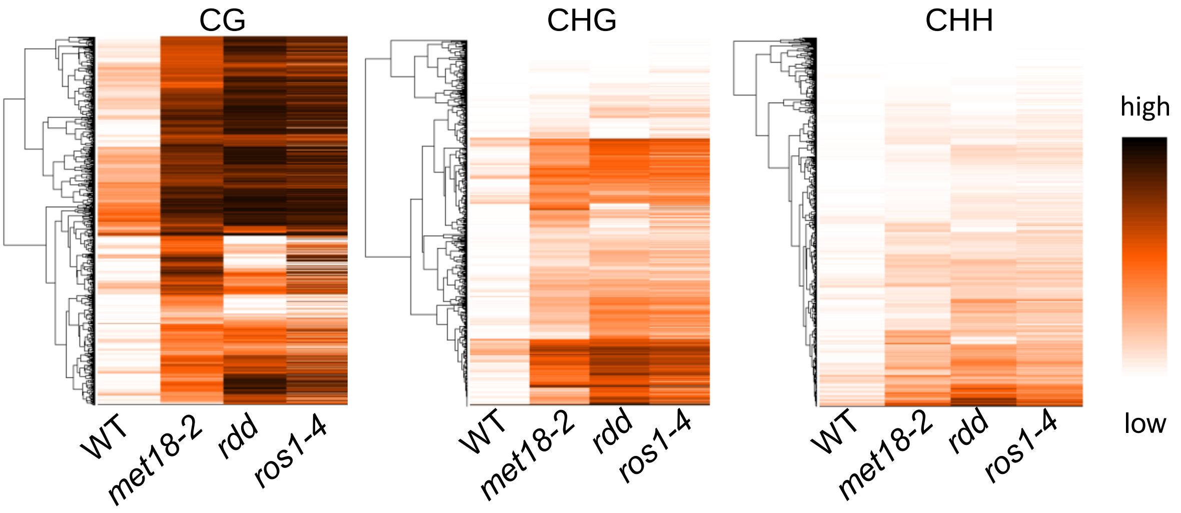

f

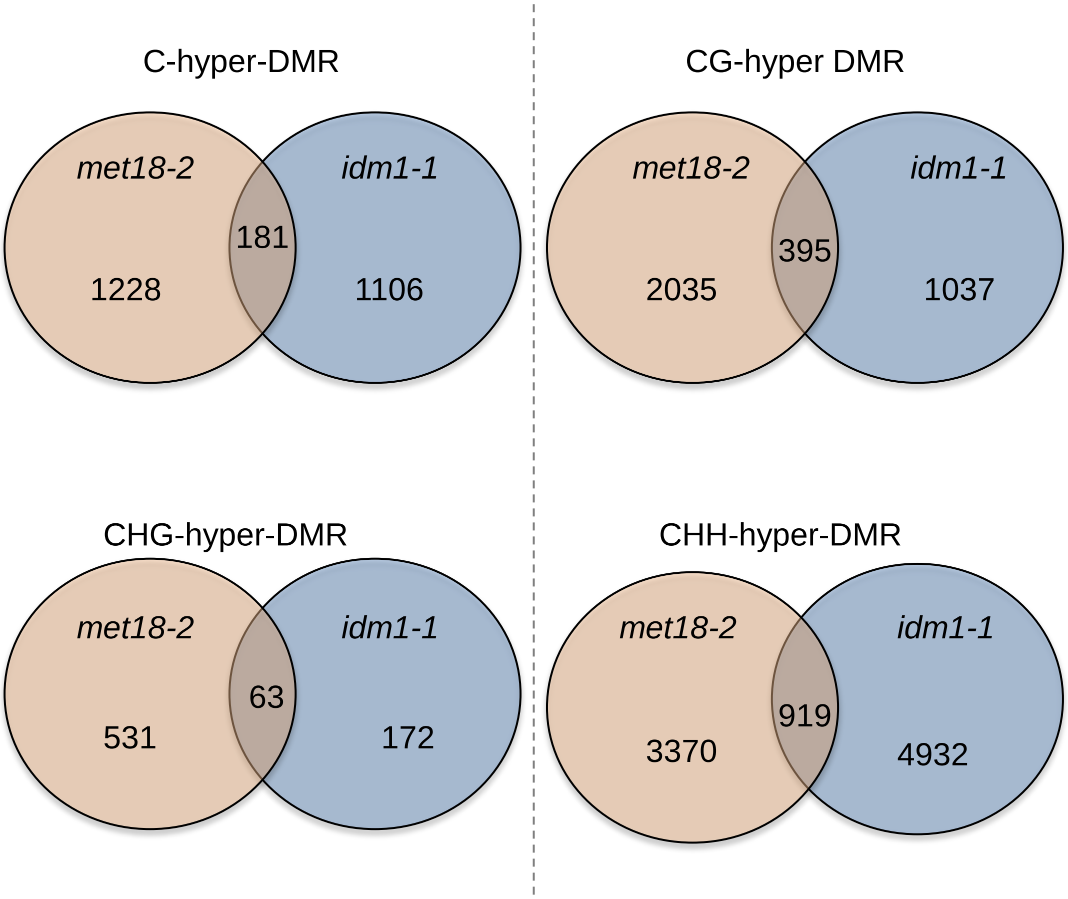

Supplementary Figure 2 continued

Supplementary Figure 2. DNA methylome analysis by whole-genome bisulfite sequencing.

(**a**) Snapshot in the Integrated Genome Browser showing the DNA methylation levels of the FAD-binding berberine gene family in WT, *ros1-4* and *met18-2*. Hypermethylated regions in the mutants are highlighted with green boxes. (**b**) Confirmation of the findings of the whole-genome bisulfite sequencing by chop-PCR using methylation-sensitive enzyme *Mcr*BC in *met18*. ACTIN serves as a control. (**c**) Distribution of hypermethylated loci on the five chromosomes in *met18-2, ros1-4* and *rdd.* (**d**) Composition of the hypermethylated loci in *met18-2, ros1-4* and *rdd.* (**e**) Heat-map showing the methylation levels of *ros1-4* and *rdd* in those regions that are hypermethylated in *met18-2* in three different contexts. The color bar is presented at right . Light yellow indicates low methylation and black indicates high methylation. (**f**) Venn diagrams showing the numbers of hyper-DMRs that either overlap between or are unique in *met18-2* and *idm1-1* in different sequence contexts.

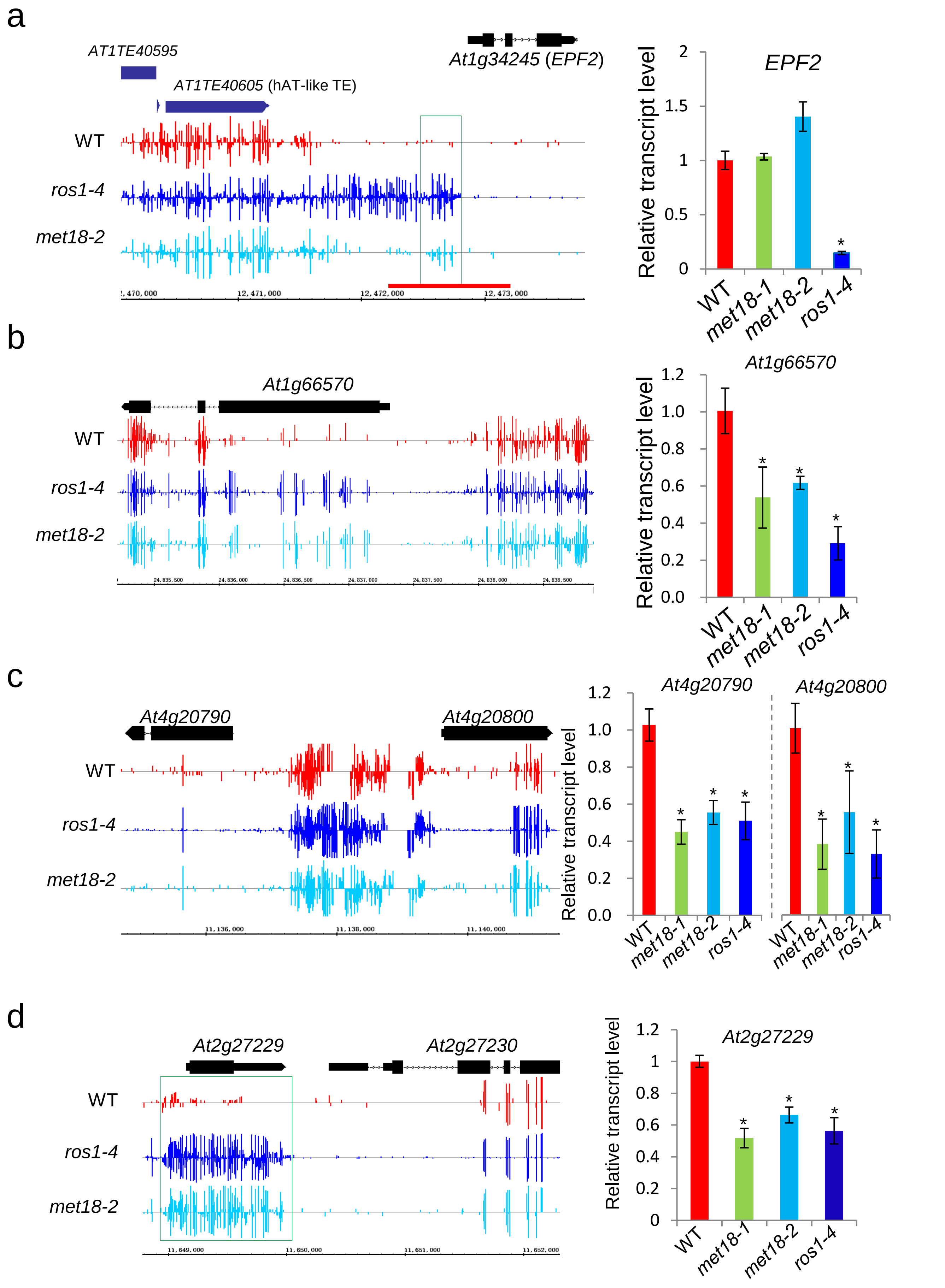

Supplementary Figure 3

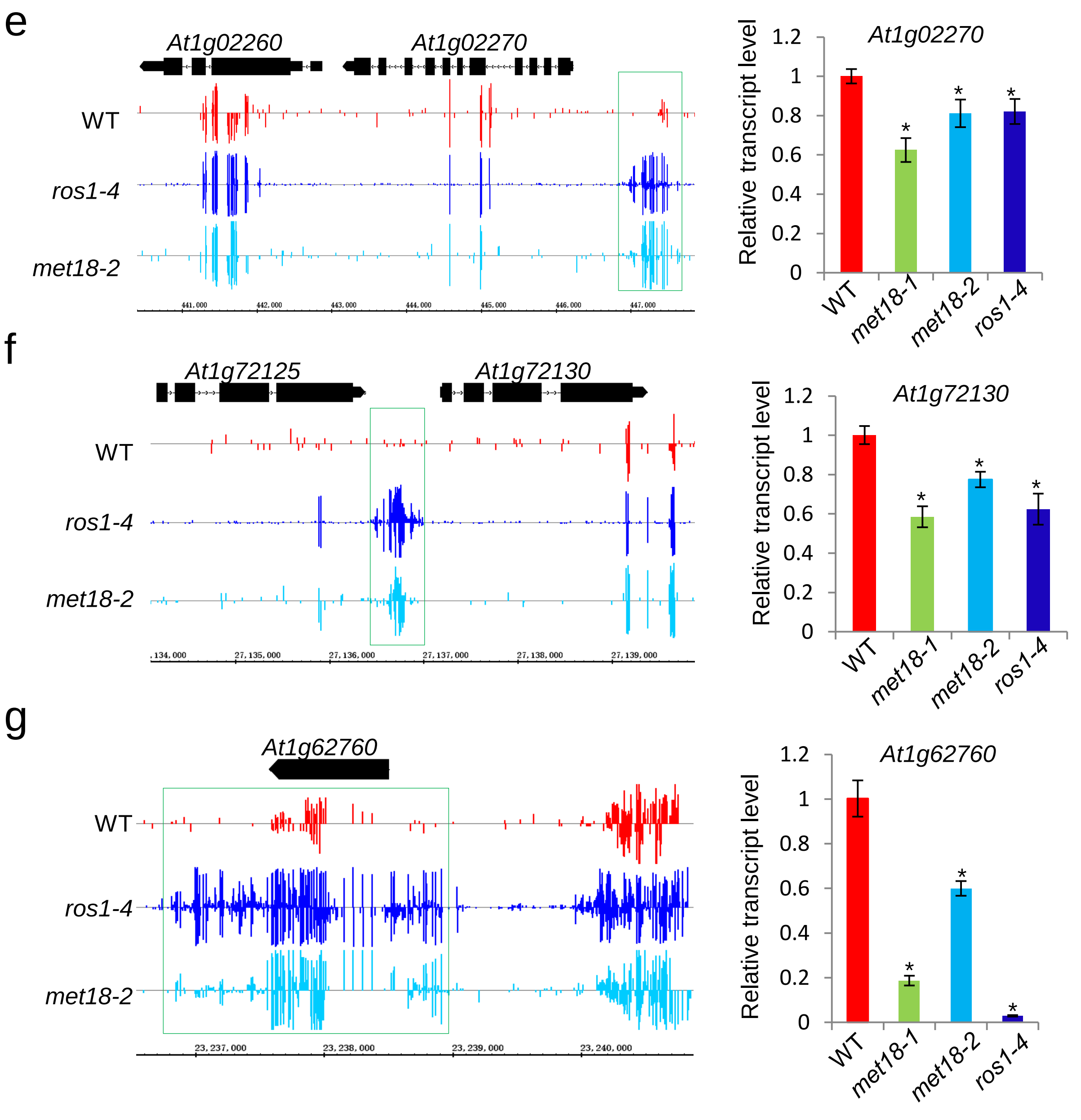

Supplementary Figure 3. Snapshots in the Integrated Genome Browser showing DNA hypermethylation in *met18-2* and *ros1-4* and the effect of the mutations on expression of the hypermethylated genes or the adjacent genes.
(**a**) Methylation status of the *EPF2* promoter in *met18-2*, *ros1-4* and WT plants. Left panel: Snapshots in the Integrated Genome Browser showing the DNA methylation levels of the *EPF2* promoter and upstream region in the WT and mutant plants. The chop-PCR marker region is highlighted with horizontal red line. Right panel: Gene expression levels in the mutants as determined by real-time PCR. (**b-g**) Methylation status and the effect of mutations on the expression of *At1g66570* (**b**), *At4g20790* and *At4g20800* genes (**c**), *At1g27229* (**d**), *At1g02270* (**e**), *At1g72130* (**f**), *At1g62760* (**g**) in the WT and mutants plants. Left panel: Snapshots in the Integrated Genome Browser showing DNA methylation levels in the mutants. Right panel: Gene expression levels in the mutants as determined by real-time PCR. *TUB8* serves as an internal control. Error bars represent standard error (n=3), $^{*}P < 0.05$. Hypermethylated regions are highlighted with green boxes.

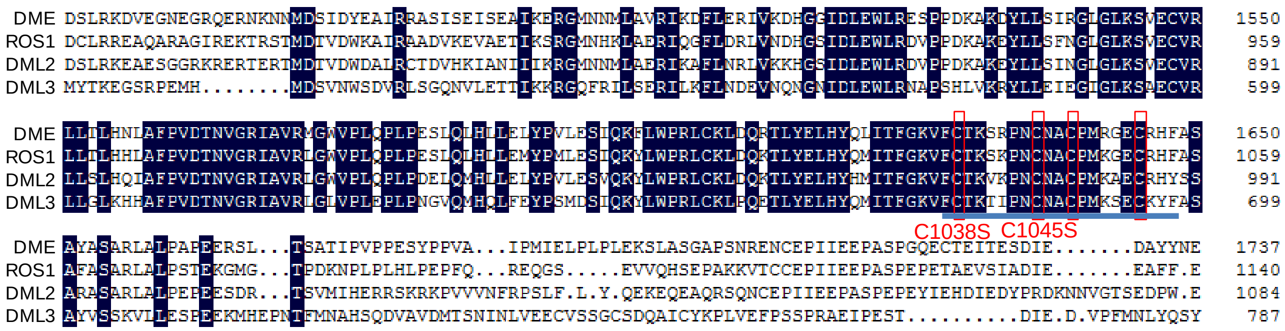

Supplementary Figure 4. Multiple sequence alignment for the HhH-GDP domains from ROS1/DME family proteins.

Conserved residues are highlighted in dark and Fe-S cluster binding motifs are labeled with a blue horizontal line. Conserved cysteine residues for Fe-S cluster binding are marked by red boxes.

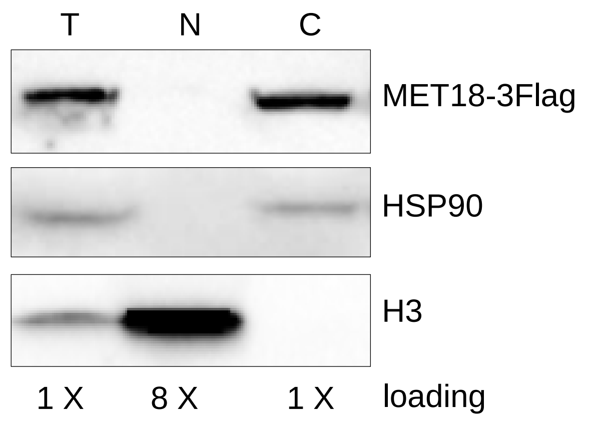

Supplementary Figure 5. Western blot analysis for subcellular localization of MET18.

After nuclear-cytoplasmic fractionation, MET18-3Flag fusion protein was detected using the anti-Flag antibody. HSP90 and histone H3 were used as markers for cytoplasmic proteins and nuclear proteins, respectively. T: total proteins, C: cytoplasmic proteins, N: nuclear proteins. N fraction loading is 8-fold than T and C fraction loading.

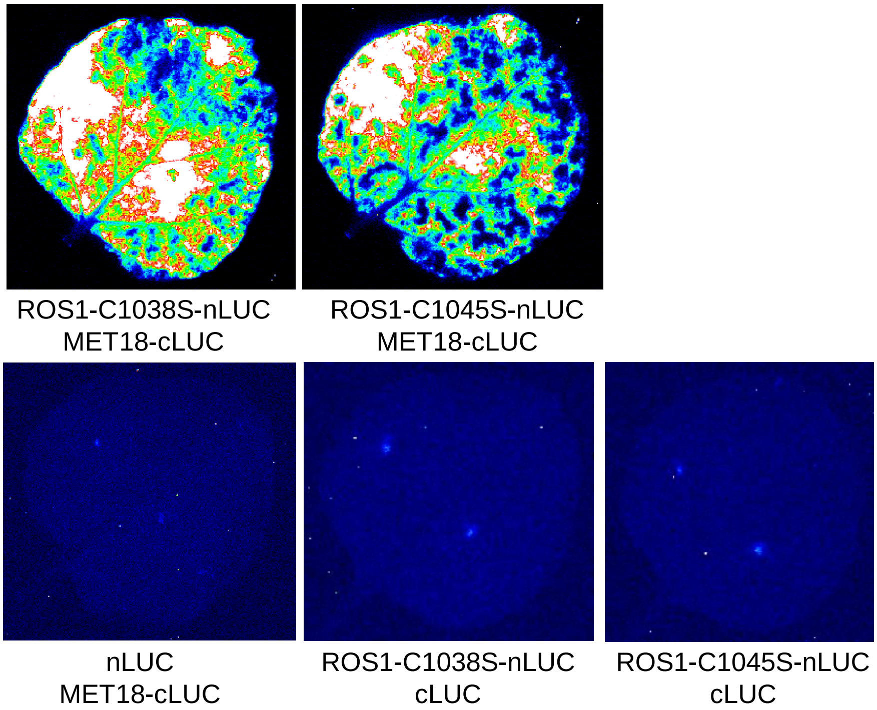

Supplementary Figure 6. Interactions between MET18 and mutated ROS1. Interactions of MET18 with ROS1 mutants as demonstrated by firefly luciferase complementation imaging assay in *Nicotiana benthamiana* leaves.

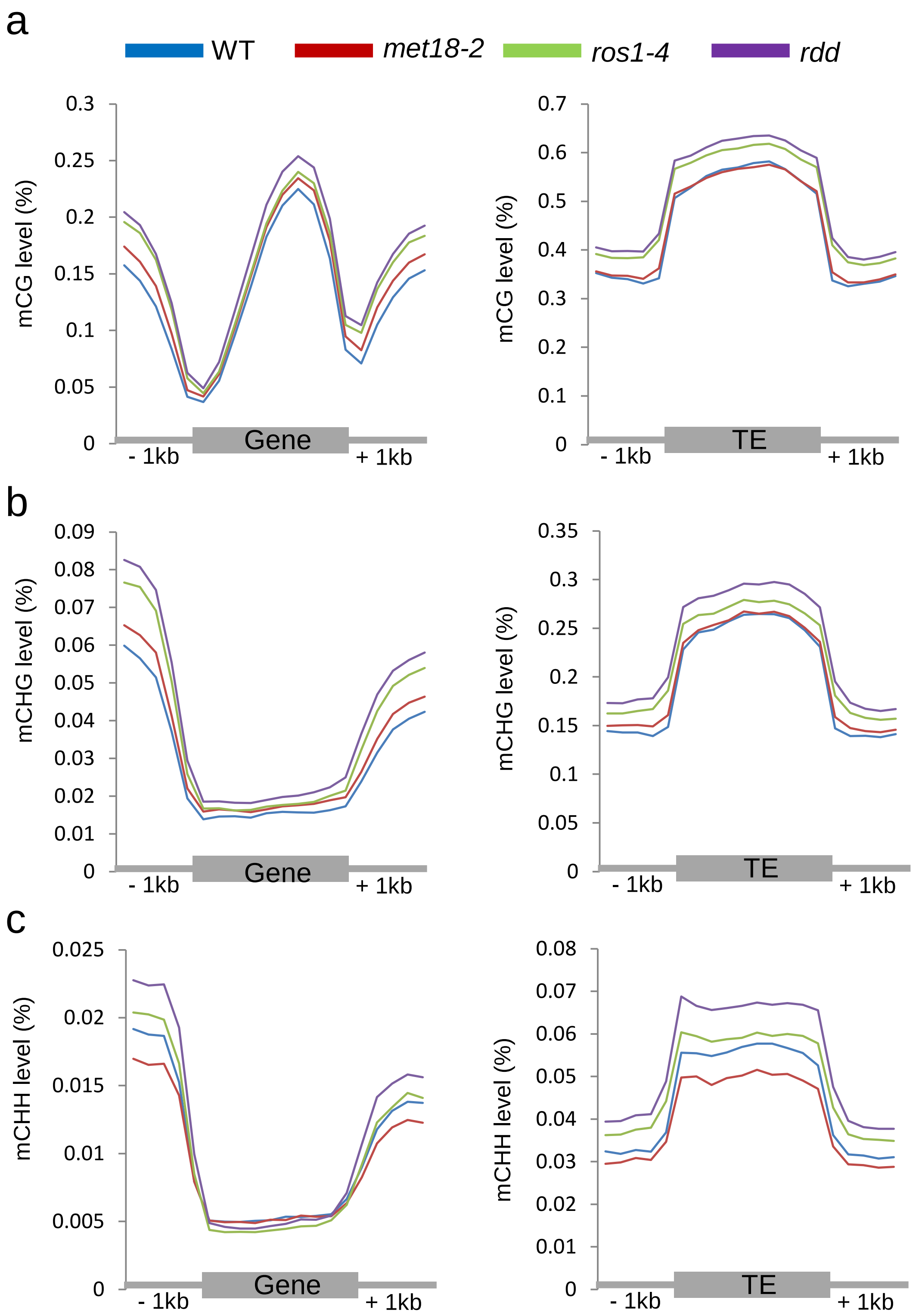

Supplementary Figure7

d

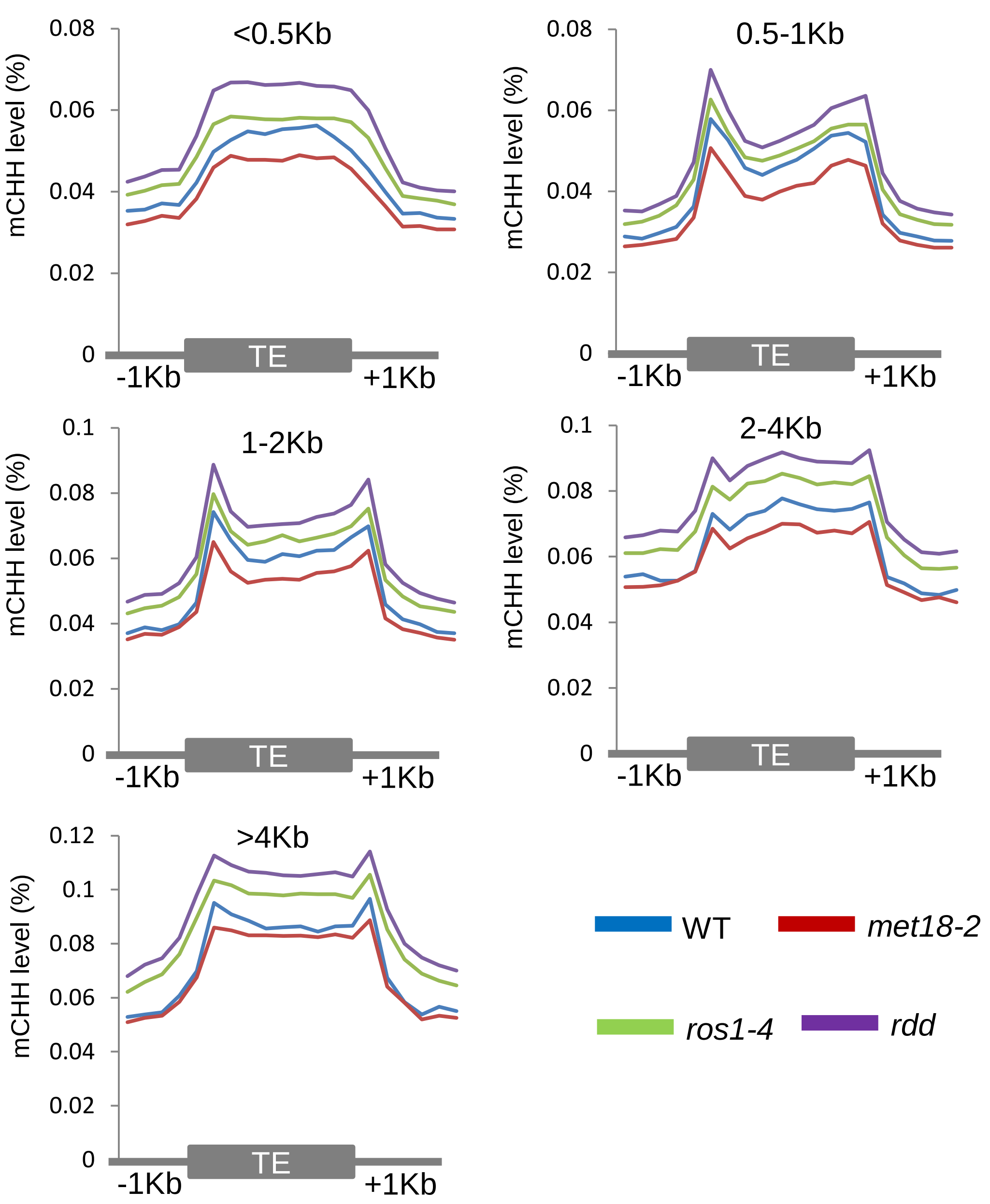

Supplementary Figure 7. DNA methylome analysis by whole-genome bisulfite sequencing. Average cytosine methylation levels in gene and TE bodies in contexts of (**a**) CG, (**b**) CHG and (**c**) CHH. Genes or TEs were aligned from 1 kb upstream of the transcription start sites to 1 kb downstream of the transcription termination sites. (**d**) Average methylation levels in the CHH context in the TEs of different length.

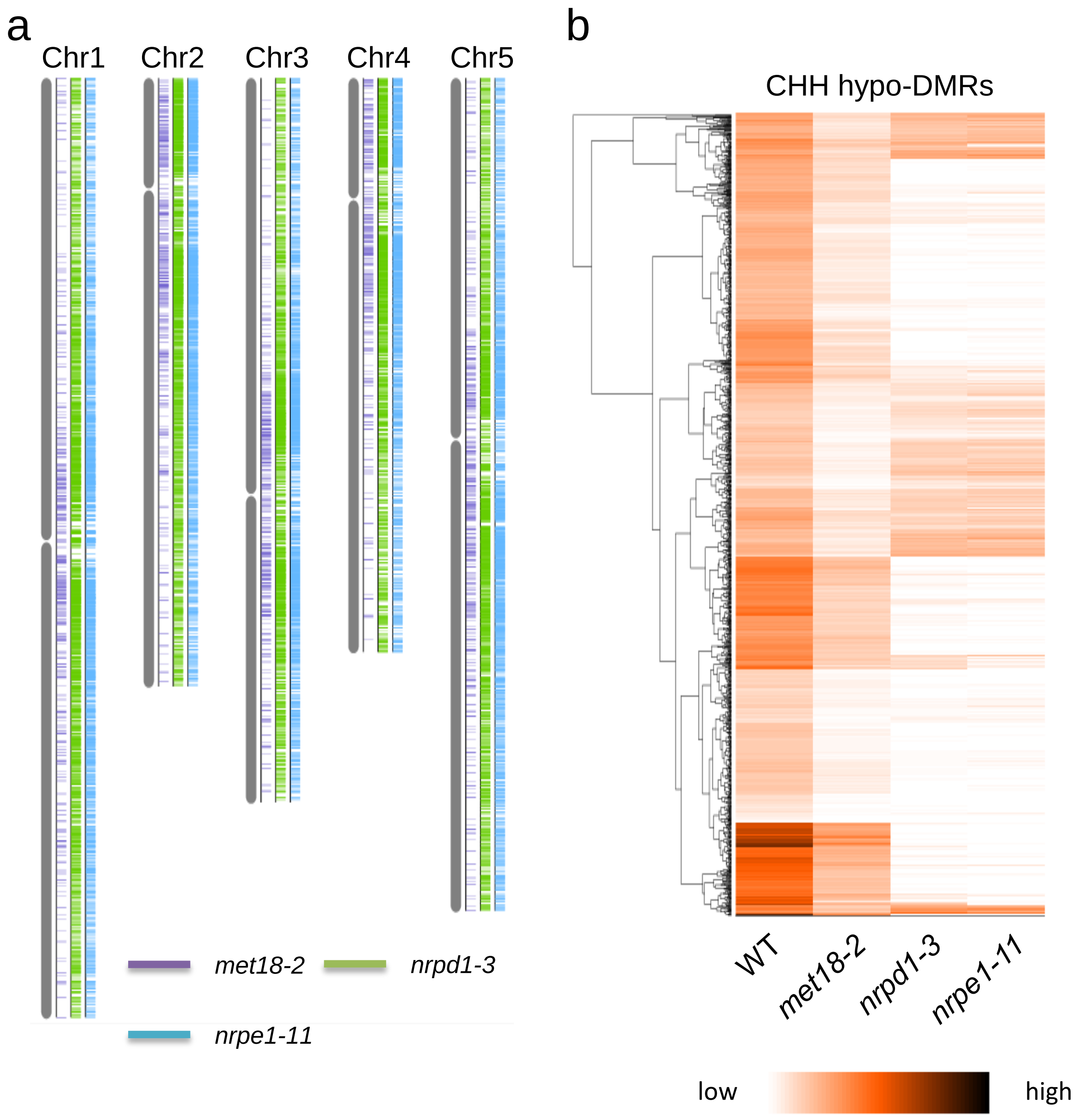

Supplementary Figure 8. Analysis of the CHH hypo-DMRs in the *met18-2* mutant. (**a**) Distribution of the CHH hypo-DMRs on the five chromosomes in *met18-2, nrpd1-3* and *nrpe1-11.* (**b**) Heat-map showing methylation levels of *nrpd1-3* and *nrpe1-11* in the regions that are hypomethylated in *met18-2* in the CHH context. The color bar is presented at bottom. Light yellow indicates low methylation and black indicates high methylation.

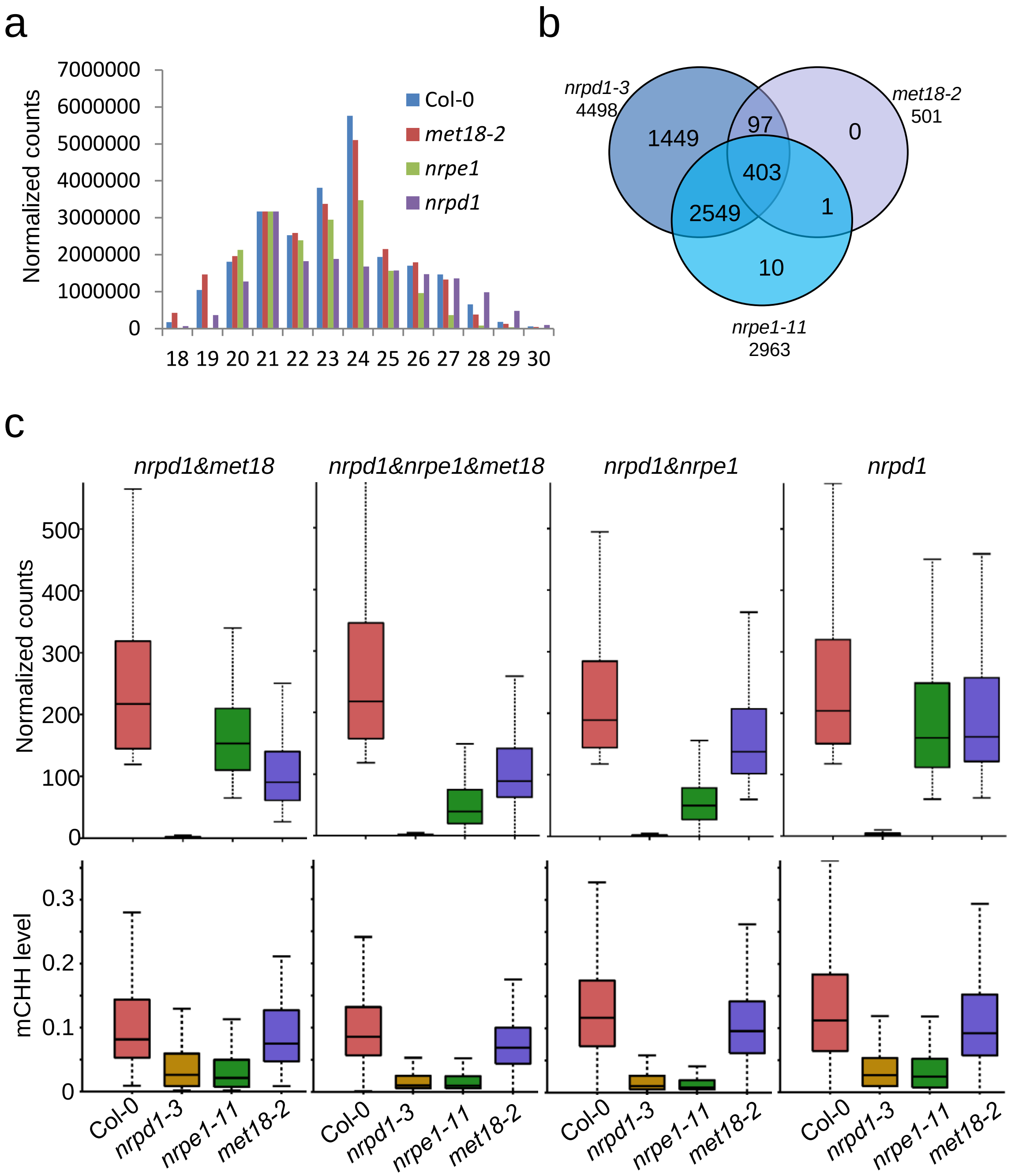

Supplementary Figure 9. The effect of *met18-2* on 24-nt siRNAs.
(**a**) Length distribution of the small RNAs. Small RNA counts were normalized to 21-nt small RNAs in each library. (**b**) Venn diagram showing the numbers of differentially expressed 24-nt siRNA clusters that overlap between and are unique in *met18-2*, *nrpd1-3*, and *nrpe1-11*. (**c**) Boxplots showing comparisons of siRNA levels and average CHH methylation levels of siRNA clusters in each subgroup in (**b**).

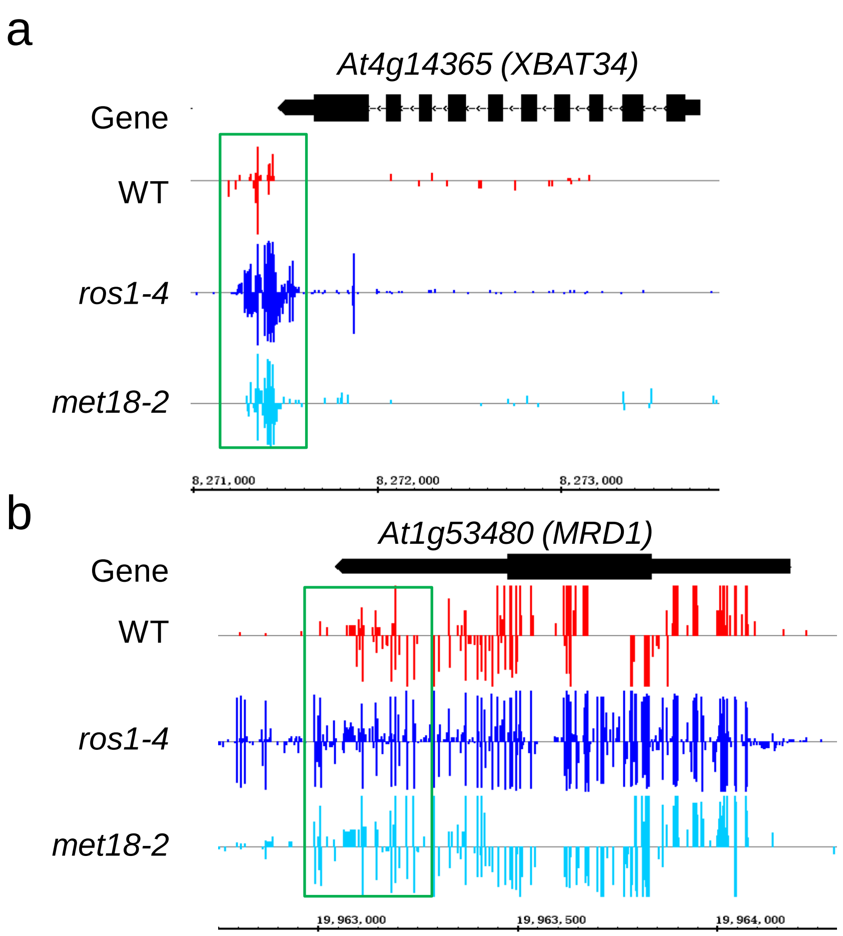

Supplementary Figure 10. Examples of whole genome bisulfite sequencing results.

(a-b) Snapshots in the Integrated Genome Browser showing total DNA methylation levels of *XBAT34* (a) and *MRD1* (b) in WT, *ros1-4* and *met18-2*. Hypermethylated regions are highlighted with green boxes.

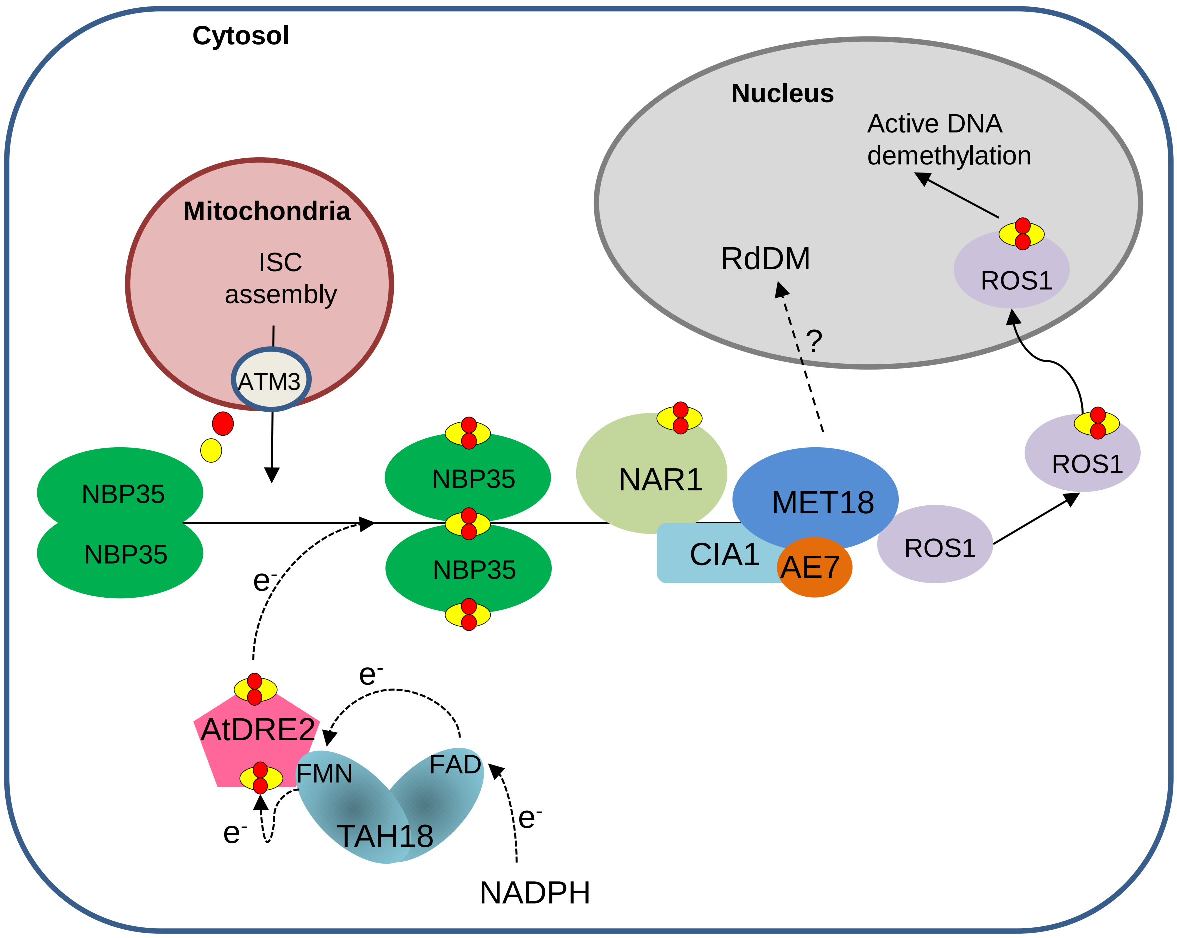

Supplement Figure 11. Working model for the MET18 regulated active DNA demethylation in *Arabidopsis*.

An unknown sulfur compound is transferred from mitochondria to the cytoplasm, followed by Iron-Sulfur cluster assembly on the CIA complex. MET18 interacts with ROS1 and helps transferring the Iron-Sulfur cluster to ROS1, which gets transported into the nucleus to accomplish active DNA demethylation process. MET18 can also affect the RdDM pathway via an unknown mechanism.
